# Organ‐specific terpenoid responses in *Tanacetum vulgare* are chemotype‐dependent

**DOI:** 10.1111/plb.70208

**Published:** 2026-04-01

**Authors:** H. Newrzella, R. Heinen, C. M. Villasante, I. Zimmer, B. Weber, P. Kary, G. Gerl, A. Neuhaus, A. Sigalas, L. Ojeda‐Prieto, J. B. Winkler, W. W. Weisser, J.‐P. Schnitzler

**Affiliations:** ^1^ Research Unit Environmental Simulation Helmholtz Munich Neuherberg Germany; ^2^ Terrestrial Ecology Research Group, Department of Life Science Systems, School of Life Sciences Technical University of Munich Freising Germany; ^3^ Animal Ecology, Institute of Biochemistry and Biology University of Potsdam Potsdam Germany; ^4^ Biodiversity Research/Systematic Botany Group, Institute of Biochemistry and Biology University of Potsdam Potsdam Germany; ^5^ Ecosystem Physiology, Faculty of Environment and Natural Resources University Freiburg Freiburg Germany

**Keywords:** *Agriotes*, chemotypes, induced defence, *Macrosiphoniella tanacetaria*, monoterpenoids, root architecture, sesquiterpenoids, *Tanacetum vulgare*

## Abstract

- Specialized metabolites such as terpenoids are known to mediate plant defence mechanisms. However, how terpenoid diversity governs inducible chemistry across organs remains poorly understood.
- We used three shoot terpenoid chemotypes of common tansy (*Tanacetum vulgare*) to test whether (i) root‐chewing wireworms induce root terpenoids locally and alter shoot terpenoids systemically and (ii) phloem‐feeding aphids induce a chemical response depending on chemotype, (iii) chemotypes differ in root‐system development. Plants were grown in rhizotrons and monitored for 60 days. After root establishment, they were challenged with root‐chewing wireworms (*Agriotes* spp.) and/or phloem‐feeding aphids (*Macrosiphoniella tanacetaria*).
- Wireworms increased stored root sesquiterpenoid levels by more than twofold in chemotypes 1 and 2, whereas chemotype 3 was largely unresponsive. Aphids did not alter root terpenoids, but significantly increased shoot monoterpenoid emissions in chemotype 1 without affecting stored pools. Therefore, storage and emission were decoupled, and induction depended on both organ (roots *versus* shoots) and chemotype. Chemotype 1 also exhibited larger root systems than the others, consistent with its stronger inducibility. Our analysis reveals a compartmentalized, chemotype‐specific defence strategy in tansy.
- This organ‐specific separation of induction pathways suggests coordinated genetic control of terpenoid biosynthesis and release, and provides a phytochemical framework for understanding above‐ *versus* belowground herbivore interactions.

## Graphical abstract

Common tansy varieties defend differently: some boost root chemicals against soil pests, while others release stronger leaf scents against aphids.

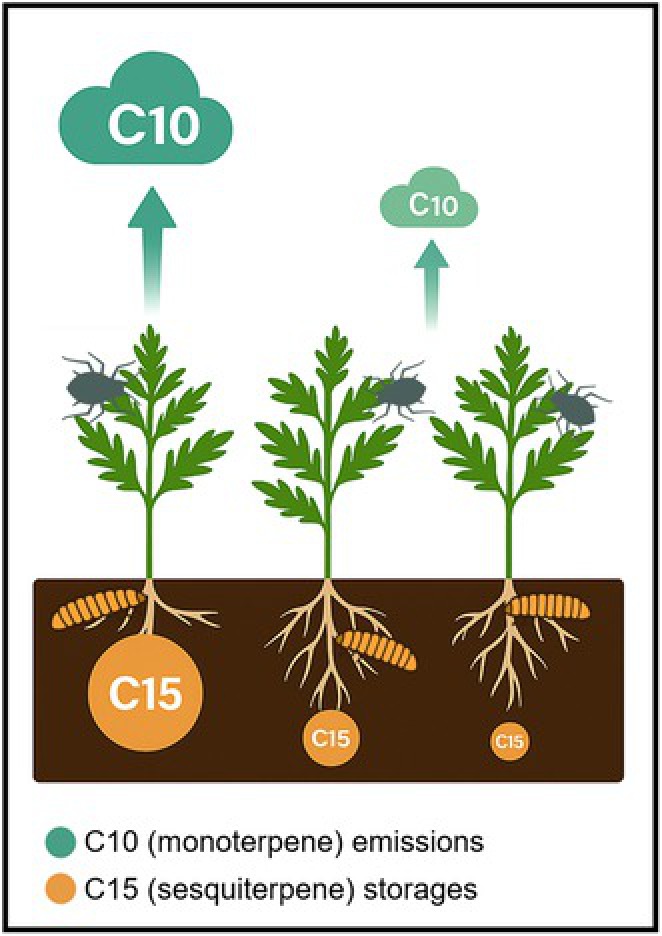

## INTRODUCTION

The plant kingdom produces a massive range of specialized metabolites that convey chemical information among plants, insects, microorganisms and adjacent roots (Massalha *et al*. 2017; Erb & Reymond 2019). These metabolites function in defence, prime uninjured tissues and neighbouring plants, regulate rhizosphere microbial populations and affect plant fitness across ecological levels (Frost *et al*. 2008; Heil & Karban 2010; Kong *et al*. 2021).

Terpenoids, specifically monoterpenoids (C_10_) and sesquiterpenoids (C_15_), constitute a major fraction of this chemical diversity in plants (Gershenzon & Dudareva 2007; Tholl 2015). Mono‐ and sesquiterpenoids are synthesized from geranyl diphosphate and farnesyl diphosphate, respectively, via reactions catalysed by terpenoid synthases (TPSs). These enzymes can convert a single substrate into multiple products, greatly expanding the structural diversity of plants (Tholl 2015; Christianson 2017). Upon their formation, terpenoids serve multiple roles in defence and many of them exhibit direct toxic or deterrent effects against herbivores and pathogens (Gershenzon & Dudareva 2007). Herbivore‐induced terpenoid blends emitted from damaged foliage may also provide an indirect line of defence by attracting predators and parasitoids to the affected plants (Turlings *et al*. 1990; Aartsma *et al*. 2017). A similar concept also applies to the belowground environment. For instance, maize roots under larval attack release the sesquiterpenoid (E)‐β‐caryophyllene, which in turn attracts entomopathogenic nematodes that infest the larvae and thereby mitigates damage from root‐feeding beetles (Rasmann *et al*. 2005; Degenhardt *et al*. 2009). Terpenoids can be released from tissues via two mechanisms. They may be actively emitted by plants, with emissions often enhanced under abiotic and biotic stress, or they can also be released following disruption of reservoir glands (Loreto & Schnitzler 2010; Niinemets *et al*. 2013). In general, terpenoids are stored in reservoir glands found in most plant species (Lange & Turner 2013). For instance, a species like *Tanacetum vulgare* L. possesses glandular trichomes in which they accumulate terpenoids in shoot tissues (Guerreiro *et al*. 2016). Likewise, many *Lamiaceae* (mint family) species have abundant peltate glandular trichomes whose essential oils are largely terpenoids (Schuurink & Tissier 2020). In contrast, precise storage sites of terpenoids in roots are still poorly resolved for many species. Among the few known storage locations are the lactifers in the roots of dicots such as dandelion that sequester defensive sesquiterpene lactones (Huber *et al*. 2016). Terpenoids stored in shoot tissues exhibit significant intraspecific variation and can be used for classifying plant chemotypes. Chemotypes are typically characterized by a single dominant compound or by multiple combinations that do not exhibit distinct dominant substances, resulting in mixed chemotypes (Clancy *et al*. 2016; Neuhaus‐Harr *et al*. 2024; Rahimova *et al*. 2024). Chemotype composition has also been connected to various ecological functions in plant‐insect interactions, including defence strategies. For instance, shoots in tea‐tree *Melaleuca alternifolia* (Maiden & Betche) Cheel plants are classified into terpenoid chemotypes, distinguished by the predominance of cineole, terpinolene and terpinen‐4‐ol (Bustos‐Segura *et al*. 2015). The same study additionally revealed that *Faex* sp. larvae grew faster on cineole‐rich plants, *Potamotrygon tigrina* M. R. de Carvalho, Sabaj Pérez & Lovejoy adults damaged terpinolene‐rich plants less and cineole‐rich plants were more susceptible to myrtle rust *Puccinia psidii* G. Winter. These results show that within species terpenoid chemotypes might shape plant–herbivore and plant–pathogen interactions aboveground. By contrast, far less is known about terpenoid storage sites and its dynamics and roles in roots, partly because belowground volatiles are hard to sample and quantify in soil, and microbial turnover can confound measurements (Delory *et al*. 2016). Nevertheless, a few studies demonstrate ecological functions of root terpenoids. For instance, in cotton (*Gossypium herbaceum* L.), terpenoid‐aldehydes are induced in roots after herbivory damage (Bezemer *et al*. 2004); in common dandelion (*Taraxacum officinale* L.), a sesquiterpene lactone in root latex deters cockchafer larvae (*Melolontha melolontha* L.) and improves plant fitness (Huber *et al*. 2016). In *Centaurea stoebe* L., root sesquiterpenoid emissions have beneficial effects on the germination and growth of neighbouring plants (Gfeller *et al*. 2019). Furthermore, exposure to *C. stoebe* root volatile organic compounds (VOCs) has been linked with altered carbohydrate and total protein levels in neighbouring *T. officinale* roots, as well as increased growth of the root herbivore *M. melolontha*. This effect is partly mimicked by (E)‐β‐caryophyllene (Huang *et al*. 2019). These examples suggest that root terpenoids can be ecologically consequential, yet comprehensive datasets linking genotype‐specific root terpenoid profiles to herbivore impacts remain scarce. At a mechanistic level, the biosynthesis of many inducible terpenoids is jasmonate‐regulated (Tholl 2015). However, phloem‐feeding aphids typically do not activate JA signalling in roots (Karssemeijer *et al*. 2020). Accordingly, foliar aphid attack is not expected to elicit a systemic increase in root sesquiterpenoids. By contrast, aphids can cause modest, chemotype‐dependent rises in foliar monoterpene emissions (Clancy *et al*. 2020).

Chemical diversity in plants defines not only chemotype profiles but can also be associated with growth traits. For instance, Ojeda‐Prieto *et al*. (2024) documented that in common tansy (*T. vulgare*), foliar terpenoid chemotypes were associated with early differences in growth that converged over the season, whereas chemotype effects on reproduction (flower‐head number and flowering phenology) persisted, linking terpenoid chemistry to reproductive performance. Comparably, in *Cinnamomum camphora* (L.) J. Presl, predefined leaf‐oil chemotypes (linalool, eucalyptol, camphor and borneol) differed in shoot morphology and photosynthetic performance. Linalool and eucalyptol chemotypes had larger leaves, and eucalyptol and camphor chemotypes showed higher photosynthetic rates (associational differences among chemotype groups, Luo *et al*. 2021). Root metabolomic composition has also been associated with root architecture in crops (Ghorbanzadeh *et al*. 2023). Because comparable datasets for terpenoid‐rich species remain scarce, we test whether root system architecture varies among chemotypes.

To study inducible chemistry in above‐ and belowground systems and root development in terpenoid‐rich species, we used tansy (*T. vulgare*), a species whose individuals differ strikingly in terpenoid composition and can therefore be assigned to distinct chemotypes. These chemotypes have so far been defined mainly by monoterpenoids, which are abundant in shoots and are known to influence interaction between tansy and associated insects (Clancy *et al*. 2016; Neuhaus‐Harr *et al*. 2024; Neuhaus‐Harr *et al*. 2025). Our recent work demonstrates that sesquiterpenoids are also key components of tansy chemistry and may serve different ecological functions (Rahimova *et al*. 2024). For example, earlier we demonstrated sharp organ‐specific sesquiterpenoid profiles across shoots, rhizomes, coarse roots and fine roots in tansy (Rahimova *et al*. 2025). Furthermore, root application of pipecolic acid (a systemic acquired resistance cue) strongly induced multiple root sesquiterpenoids, significantly more than in shoots. Yet whether root sesquiterpenoid chemotypes are similarly inducible under real herbivory remains unknown. To fill that gap, we exposed three chemotypes to both herbivores in sequence, root‐chewing wireworms (*Agriotes* spp.) followed by the phloem‐feeding aphid (*Macrosiphoniella tanacetaria* Kaltenbach) and quantified terpenoid stores and emissions from shoots and roots to test how cross‐guild attacks influence chemotype‐dependent defences above‐ and belowground. We asked the following questions:

1. Do both wireworms and aphids, applied sequentially, trigger localized *versus* systemic shifts in terpenoid stores in tansy roots and shoots, and are the magnitudes of these shifts chemotype‐specific?
2. Does feeding by the aphids, either alone or together with wireworms, change terpenoid emission in shoots and are these changes chemotype‐specific?
3. Do plant phenotype traits, specifically root system architecture, differ depending on the chemotype identity in tansy?

## MATERIAL AND METHODS

### Plant material

Three *T. vulgare* L. chemotypes were selected from a stock of chemotypes available at the Terrestrial Ecology Research Group, and originally sourced from seeds collected in Jena, Germany. For further details of the origin of the chemotypes and thus plant materials, please see Neuhaus‐Harr *et al*. (2024). All plants were propagated vegetatively via stem cuttings taken from plants at the end of summer prior to the experiment which took place in autumn.

### Experimental set‐up

In order to assess the phenotypic development of tansy and the effect of above‐ and belowground treatments in a non‐invasive approach, we used rhizotrons in a high‐throughput phenotyping (HTP) facility (Fitness‐SCREEN) at Helmholtz Munich. Two‐week‐old plants from three distinct chemotypes were transferred to the designated rhizotrons (40 cm × 80 cm × 4 cm; length × height × width) containing a peat substrate (Einheitserde Classic type CL‐T, Patzer Erden GmbH, Sinntal, Germany) and black basalt sand mixture (1/1.2, v/v). The plants were cultivated under controlled conditions, with a 14‐h photoperiod (light:dark) at 20°C:18°C and 40%:60% relative humidity (day:night). The illuminance of the plants was based on the radiation intensity outside the greenhouse. If the outside measurement was less than 30 klx (~550 μmol m^−2^ s^−1^ PPFD) during daytime, the greenhouse's lamp ceiling consisting of an equal mixture of MGR‐K‐400‐CHD‐Watt and MGR‐K‐400‐SOD lamps (DH Licht GmbH, Wülfrath, Germany) was switched on between 05:00 and 19:00. During the supplementary lighting phases, the light intensity at canopy height was ~220 μmol m^−2^ s^−1^ PPFD.

Prior to the treatment, each plant was watered twice a week with 200 mL of the diluted (1/100, v/v) fertilizer Hakaphos® Rot (8% N, 12% P_2_O_5_, 24% K_2_O, 4% MgO, 31% SO_3_, 0.01% B, 0.02% Cu, 0.05% Fe, 0.05% Mn, 0.001% Mo, 0.02% Zn; Compo Expert GmbH, Münster, Germany). Overall, we transplanted 60 plants (one per rhizotron) into individual rhizotrons positioned on automated carriers. Each chemotype comprised three original source plants (genotypes), which were propagated clonally by stem cuttings to produce the experimental plants here (n = 20 plants per chemotype). Chemotype identity was randomized across carriers.

### Herbivory and insect colonies

The aboveground treatment involved the use of aphids as phloem‐feeding herbivores. The aphid species, *M. tanacetaria*, were obtained from the Terrestrial Ecology Research Group at TUM and originally collected from plants in Jena, Germany. The aphids were reared on fresh tansy leaves, which were collected from different lines of each of six chemotypes used in Neuhaus‐Harr *et al*. (2025): Bthu_High and Bthu_Low (β‐thujone dominant), Athu_Bthu (α−/β‐thujone dominant), Chrys_Acet (trans‐chrysanthenyl acetate dominant) and Mixed_High and Mixed_Low (mixed chemotypes with multiple major compounds). The leaf origins were randomly picked to avoid aphids developing affinity to one specific chemotype. The aphids were reared under controlled conditions (photoperiod: 14 h:10 h; temperature: 21°C:17°C; day:night) and were used as a standardized elicitor rather than for performance assays. For the plant treatment, age‐specific cohorts of aphids were established to maintain comparable population growth across the different plants during the experiment. For this, 50 adult aphids were selected from the main colony and kept on clipped tansy leaves in a petri dish (150 mm in diameter and 15 mm in height) for 24 h. After removing the adult aphids, the nymphs were allowed to grow for an additional 2 days and these 2 days old nymphs were used for the aphid treatment.

For the belowground treatment, wireworms, which are known to feed on plant roots, were used. The larval population was collected in the fields, and hence consisted of a mixture of *Agriotes lineatus* L. and *Agriotes obscurus* L. obtained from Wageningen University in the Netherlands. These larvae were reared in covered soil containers with commercially available potatoes until treatment.

### Treatment

We initiated the treatment 7 weeks after propagation of the plants. The experimental design of our treatment process is provided in Fig. S1. The treatment groups included control plants with no treatment, plants treated only with wireworms, plants treated only with aphids and plants that had been treated with both wireworms and aphids. The selection and introduction of worms was conducted in two steps. In the first step, we opened the rhizotrons and randomly placed five worms within the rhizotrons. In the second step, we dug small holes in the soil closer to the stem and introduced the next batch of four worms (in total, nine worms per plant) to the soil. We continuously monitored each plant to ensure that worms did not escape and were contained within the rhizotrons. Because tansy is an aromatic plant sensitive to minimal disruption, we used standardized handling steps across treatments to minimize disturbance.

Ten days after worm treatment, 10 aphid nymphs were carefully applied to one mature leaf of the plants designated for aphid and worm and aphid treatments and covered with sheer organza bags (9.5 × 15 cm, length × width) to prevent potential escape. After 19 days of the aphid treatment, root and shoot tissue were harvested and frozen in liquid nitrogen. Samples were kept at −70°C until further chemical analyses. During the final harvest, the number of aphids per leaf was counted and the wireworms were retrieved, except for two that were found dead. At the final harvest, we also assessed the natural infestation level by thrips, powdery mildew and *Coloradoa tanacetina* Walker that occurred in the greenhouse during the experiment so that their impacts could be accounted for in the statistical analyses.

### Hexane extraction of terpenoids and GC–MS analyses

For the solvent extraction of stored compounds, shoot tissues were extracted from 59 plants (chemotype 1: n = 20; chemotype 2: n = 19; and chemotype 3: n = 20), as well as root tissues from 58 plants (chemotype 1: n = 19; chemotype 2: n = 20; and chemotype 3: n = 19). Three samples (one shoot and two roots) could not be extracted due to the absence of the required material. In the laboratory, frozen shoot and root samples were ground to a fine powder under cold conditions. The shoot tissue was pulverized using a Silamat S6 mixing device (Ivoclar Vivadent AG, Liechtenstein) in 10‐s intervals, repeated three to four times until a fine powder was obtained. As root tissue is more fibrous, the roots were pre‐ground manually using a mortar and pestle before being further homogenized with the Silamat S6 in 10‐s intervals, repeated 5–6 times. All powders were stored at −70°C until extraction. We conducted the extraction in the following steps: 300 μl hexane containing 860 pmoL μl^−1^ internal standard (IS) (monoterpenoid δ‐2‐carene) was added to approximately 150 mg frozen tissue (shoot or root) powder, the sample was then vortexed for a minute and then stored at 4°C for 24 h. After 24 h, 150 μl of the liquid extract was removed and stored at 4°C. Additional 150 μl hexane and IS mixture was added to the material, which was then vortexed and stored again at 4°C for 24 h. 150 μl of the liquid extract was again removed and combined with the previously collected extract. One microlitre of sample was injected into an empty glass cartridge containing a glass micro‐vial that was placed on the autosampler of the gas chromatography–mass spectrometer (GC–MS) system. The GC–MS temperature program and instrumental conditions were as described in Rahimova *et al*. (2024, 2025), led by the same author.

### Compound identification

Chromatographic peak integration was performed using the Agilent Enhanced ChemStation (version E.02.00.493; Agilent Technologies, Santa Clara, CA, USA) with a consistent set of integrator events applied to all samples. The initial peak width was set to 0.020 min, shoulder detection was disabled and the threshold setting in the instrument software was set to 17.0. Additionally, an initial area reject value of 1 (in the integrator's peak‐area units) was applied to exclude very small features with a very low signal from automated peak calling. The detected peaks were then tentatively identified by comparing the mass spectra of each chromatogram with those in the Mass Spectral Library (National Institute of Standards and Technology: NIST 20) and the Kovats retention indices. The latter were calculated based on the retention times of a saturated alkane mixture (C9‐C21; Sigma‐Aldrich, St. Louis, MO, USA), which was measured alongside the samples.

### Quantification

Because authentic standards and compound‐specific response factors were not available for all detected terpenoids, we used the relative quantification method where compound abundances were normalized by IS and sample mass and are reported as pmol equivalents per mg tissue. First, peaks were integrated for each detected compound $\left(i\right)$ and for the IS in every sample. Compound peak areas were then normalized to the IS peak areas:

$$n_{i}=\left(\frac{A_{i}}{A_{\text{IS}}}\right)\times n_{\text{IS}}$$

where $A_{i}$ and $A_{\text{IS}}$ are the integrated peak areas of compound $\left(i\right)$ and IS, respectively, and $n_{\text{IS}}$ is the amount of IS (860 pmol μl^−1^; constant across samples). Values were then normalized by the sample fresh weight (mg) to obtain relative concentration:

$$C_{i}=\frac{n_{i}}{m}$$

where $n_{i}$ is an IS‐normalized amount of a compound and m is the mass of fresh samples (pmol mg^−1^). Pure standards of sabinene, α‐pinene, linalool, methyl salicylate, bornyl acetate, β‐caryophyllene and α‐humulene were prepared at six concentrations (55, 120, 250, 400, 550 and 800 pmol μl^−1^) and analysed to confirm retention times, mass spectral identity and to verify signal linearity over the working range.

### VOC collection and GC–MS analyses

During the experiment, VOCs were collected from the root and shoot tissues of plants in the greenhouse using a passive sampling method with Twisters (Gerstel, Mülheim an der Ruhr, Germany) that are made of non‐polar polydimethylsiloxane‐coated stir bars (film thickness 0.5 mm, length 10 mm). Their non‐polar nature allows them to efficiently capture VOCs. Shoot VOC sampling was initiated 14 days after the onset of aphid treatment. Twisters were attached to paper clips and placed in the bushy parts of the plant canopy in the morning (between 10:00 and 11:00 h) and left in place for 36 h. Additionally, we positioned twisters in various appropriate locations throughout the greenhouse to ensure a comprehensive background measurement, allowing us to correct later for any potential peaks that might not originate from tansy. For aboveground passive VOC sampling, we analysed 58 plants (chemotype 1: n = 18; chemotype 2: n = 20; chemotype 3: n = 20). In two chemotype 1 samples, no plant VOC peaks were detected under the standardized integration settings (only the IS); these samples were therefore treated as non‐detects and not included in downstream emission analyses. When collecting VOCs from root tissues, we opened the rhizotrons' glass covers and inserted twisters into four different sections of the rhizotron and root system. The first twister was attached to the upper part of the rhizotron (approximately 5 cm from the top), where most of the coarse roots are found. The second and third twisters were attached to individual roots, which were loosely wrapped in aluminium bags (8 × 5 cm length × width) along with the twisters. These two twisters were positioned in the lower part of the rhizotron (30–40 cm from the top). For each rhizotron, a spoonful of soil was also collected in an aluminium bag together with a twister to control for potential contamination from the soil‐derived VOCs. In total, 120 twisters were deployed and subsequently analysed: 30 in the top zone (n = 10 per chemotype), 60 in the lower zone (n = 20 per chemotype; two twisters per rhizotron on individual roots) and 30 in the soil control, away from visible roots (n = 10 per chemotype). Root VOC sampling was initiated 12 days after the onset of wireworm treatment; twisters were positioned in the rhizotrons in the morning (between 09:00 and 11:00 h) and left in place for 60 h. The headspace analysis of VOC samples was conducted following the methodology described by Zhu *et al*. (2025) using thermo‐desorption gas chromatography–mass spectrometry (TD‐GC–MS; TD by Gerstel; GC: 7890A, MS: 5975C, Agilent Technologies, Palo Alto, CA, USA). Compound identification of emitted terpenoids was performed using the same approach as for solvent‐extracted (stored) terpenoids.

### Quantification

Quantification of VOCs was performed as described for the solvent (hexane) extracts. Briefly, peak areas were integrated for each compound and the IS in each TD‐GC–MS chromatogram, and compound signals were normalized to the IS according to:

$$n_{i}=\left(\frac{A_{i}}{A_{\text{IS}}}\right)\times n_{\text{IS}}$$

where $A_{i}$ and $A_{\text{IS}}$ are the peak areas of compound $\left(i\right)$ and IS, respectively. For passive VOC sampling, values were reported as IS‐normalized relative abundances.

### Phenotypical analysis

We used a non‐invasive, high‐throughput phenotyping (HTP) workflow to quantify root traits over 60 days of monitoring. First, image acquisition was performed for belowground traits. The transparent side of each rhizotron was photographed daily with fixed camera setups: Allied Vision Prosilica GT 6600 paired with a Milvus 2/50 M lens, producing 6012 × 2821‐pixel grayscale images that spanned the full 685 × 322 mm observation window (≈8765 pixels mm^−2^ resolution). Root image analysis pipeline is as follows: first, raw root images (.bmp) were converted to .tif to be compatible with RootPainter 0.2.21 (Smith *et al*. 2022), an AI‐driven segmentation tool that relies on convolutional neural networks (CNNs). Following the corrective training protocol proposed by Seethepalli *et al*. (2021), RootPainter automatically extracted one 900‐pixel tile per image. The operator iteratively annotated roots and background until model predictions stabilized, at which point training was halted and the best‐performing model was used to segment the full image set. Segmented outputs were then converted into a format readable by RhizoVision Explorer 2.0.3 for quantitative trait extraction (Seethepalli & York 2020; Seethepalli *et al*. 2021). Within RhizoVision, the ‘broken roots’ mode was selected to improve reconstruction of discontinuous root segments, and the following global filters were applied: threshold = 200, removal of non‐root objects larger than 1 mm^2^ and root‐pruning threshold = 1. Batch processing produced many features for every image and three of those features, total visible root length, root volume and root surface area, are used in this study. Shoot architectural features will not be part of this study.

### Statistical analysis

Data visualization was performed in R (version 4.3.3; R Core Team 2024) using ggplot2 (Wickham 2016) for graphics; patchwork (Pedersen 2020) to combine panels (*e.g*., boxplots); corrplot (Wei & Simko 2017) for correlation matrices; ComplexHeatmap (Gu *et al*. 2016) for heatmap. Final figures were edited and exported at high resolution in Inkscape, an open‐source vector‐graphics editor.

#### Analysis of the stored and emitted compounds

To effectively examine the primary focus of our study, we analysed total concentration of terpenoids (both mono‐ and sesquiterpenoids) in roots and shoots (stored pools) and shoot emitted terpenoids with linear models in R (base *lm*). Response variables were log‐transformed to stabilize variance, and these log‐transformed values were used for all statistical analyses and figure presentations. Unless noted otherwise, models included the unplanned biotic covariates thrips, powdery mildew and *C. tanacetina* (monitoring recorded at final harvest) and the main effect of experimental factors, wireworms (Worm), aphids (Aphid) and plant chemotype, with all two‐ and three‐way interactions among Worm, Aphid and Chemotype. Peak intensities of stored root monoterpenoids were frequently near the integration limit and were therefore not reliably detected in all samples. Non‐detects (peaks below the standardized integration threshold) were treated as missing values rather than true absence and were excluded prior to modelling, which resulted in empty cells in some chemotype × treatment combinations, yielding a rank‐deficient design (aliased, non‐estimable coefficients) for terms involving chemotype. We therefore fit a reduced model excluding chemotype and its interaction terms for stored root monoterpenoids. Similarly, emitted shoot sesquiterpenoids were low in abundance and thus detected in 35 plants out of 58 (non‐detects reflect signals below standardized integration thresholds), and thus we modelled the positive subset only. For emission data, the unplanned covariates were omitted because headspace sampling occurred 10 days before harvest (timing mismatch with the covariate measurements). Stored root sesquiterpenoids, stored shoot mono and sesquiterpenoids and emitted shoot monoterpenoids were positive across all plants and required no special handling. We used Type III anova (*car::Anova*; Fox & Weisberg 2019) with sum‐to‐zero contrasts for factors to test model terms. Model assumptions (normality and homoscedasticity of residuals) were assessed by visual inspection of Q‐Q and residual‐*versus*‐fitted plots.

**Fig. 1:**
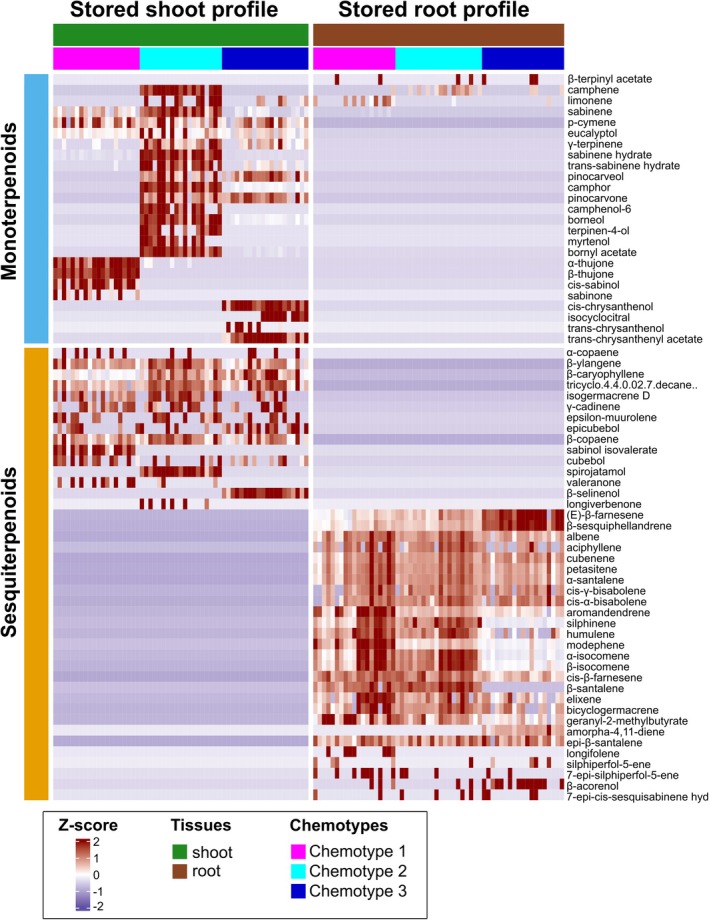
Terpenoid fingerprints of three tansy chemotypes across shoots and roots. Heatmap showing stored mono‐ and sesquiterpenoid profiles measured in shoot and root tissues of individual plants belonging to three tansy chemotypes. Each column represents one individual plant sample, ordered by tissue (shoot, root) and then by chemotype (Chemotypes 1–3), as indicated by the colour bars above the heatmap. Rows represent individual compounds and are grouped into monoterpenoids and sesquiterpenoids (left side bar and row split). Cell values are row‐wise *Z*‐scores (*z*‐scored per compound across all samples) to visualize relative differences in compound abundance among individuals. Warmer colours indicate higher relative abundance, and cooler colours indicate lower relative abundance for a given compound. Red indicates higher‐than‐average relative abundance for a given compound, white indicates approximately average abundance and purple/blue indicates lower‐than‐average relative abundance.

For the figures presented in this study, we also obtained pairwise comparisons from the full model using estimated marginal means (*emmeans*; Lenth 2024). We report: (1) all‐pairs treatment comparisons or contrasts among the four treatment combinations within a chemotype for Fig. 1 (*e.g*., Control, Worm, Aphid, Worm & Aphid) using Tukey‐adjustment; (2) simple‐effects within chemotype for Fig. 4 (*e.g*., Aphid *versus* Control within each chemotype) using *emmeans*, averaging over Worm with observed cell weights (*weights* = ‘*cells*’). *P*‐values were Bonferroni‐adjusted across the set of chemotype‐specific tests. Statistical significance was set at α = 0.05 after adjustment.

#### Analysis of root‐growth traits

We analysed surface area, total root length, volume and root:shoot ratio with generalized additive mixed models (GAMMs) fit with ‘*mgcv*’ package (Wood 2017) and smoothing parameters were estimated by restricted maximum likelihood (REML). To account for repeated measurements of the same plant, we added a plant‐level random intercept. For chemotype differences, we obtained estimated marginal means (EMMs) from the GAMMs using *‘emmeans’*, averaging uniformly over the observed day of year range. Pairwise chemotype contrasts were tested with Wald χ^2^ (df = 1) derived from the EMM contrasts, and *P*‐values were adjusted within trait using the Benjamini‐Hochberg (BH) procedure (FDR α = 0.05). We visualized trait trajectories over day of year using ‘*geom_smooth*’ method with ‘*mgcv::gam*’ (thin‐plate splines, k = 10, REML), fitting a separate smoother for each chemotype and displaying 95% confidence intervals.

## RESULTS

### Overview of stored terpenoid profiles in shoots and roots of three tansy chemotypes

The combined heatmap (Fig. 1) summarizes the stored mono‐ and sesquiterpenoid profiles in shoots and roots across the three selected chemotypes. The monoterpenoid composition in shoots was highly distinct between chemotypes (Fig. 1). Shoot tissues of chemotype 1 were β‐thujone‐dominant (with α‐thujone, cis‐sabinol and sabinone also enriched), chemotype 2 exhibited a mixed profile with camphor, sabinene hydrate, bornyl acetate and other minor constituents and chemotype 3 was characterized by strong enrichment of trans‐chrysanthenyl acetate with cis‐chrysanthenol as a secondary component. These patterns are in alignment with the identities reported for our source material of chemotypes (Athu_Bthu, Mixed_low, Chrys_Acet) by Neuhaus‐Harr *et al*. (2024, 2025). We note minor quantitative differences compared to Neuhaus‐Harr *et al*., particularly in the relative α−/β‐thujone abundance, which are plausibly attributable to differences in GC–MS methods and extraction protocols between the studies, performed in different labs (Eckert *et al*. 2023). Alternatively, quantitative variation in the α−/β‐thujone ratio could reflect underlying genetic variation (*e.g*., nucleotide substitutions affecting thujone‐biosynthetic enzymes), although this was not tested here. Importantly, the qualitative profile of each chemotype remained unchanged, supporting the same chemotype assignment. Across shoot sesquiterpenoids, β‐copaene was consistently detected and showed higher relative abundance across chemotypes (Fig. 1). Each chemotype, however, also showed a unique sesquiterpenoid, with sabinol isovalerate identified in chemotype 1, β‐selinenol in chemotype 2 and spirojatamol in chemotype 3. In addition to the strong variability of many monoterpenoids and some sesquiterpenoids between chemotypes, variability was also observed among plants within the same chemotype. For example, sabinone was only detected in chemotype 1 but was present in only 10 individuals of that chemotype. Similarly, trans‐chrysanthenol was detected in only seven individuals of chemotype 3, while the sesquiterpenoid longiverbonene was only detected in six chemotype 2 individuals. Plants exhibiting distinct mono‐ and/or sesquiterpenoid signatures within their respective chemotype have previously been classified as different clonal lineages (‘daughters’; see Neuhaus‐Harr *et al*. 2024). However, this study was not designed to test daughter‐level effects, and daughter identity was not incorporated into the full factorial analyses because the available number of individuals per daughter did not allow balanced representation across the four herbivory treatments. We therefore pooled daughters within each chemotype and treated them as biological replicates.

The stored root terpenoid profile contained only four monoterpenoid compounds, compared to a broader set of sesquiterpenoid compounds (Fig. 1). Similar patterns have been reported in previous studies (Kleine & Müller 2013; Rahimova *et al*. 2025), with tansy roots containing fewer monoterpenoids and being dominated by sesquiterpenoids. Across the stored root profile, sesquiterpenoids clearly dominated all three chemotypes (see Fig. 1), with a broad set of compounds consistently showing stronger relative intensity. Despite the overall similarity of being sesquiterpenoid‐rich, the heatmap reveals chemotype‐specific shifts in relative abundance. Notably, β‐sesquiphellandrene and (E)‐β‐farnesene were prevalent across all three chemotypes, but were more abundant in chemotype 3 than in chemotypes 1 and 2. Conversely, α‐ and β‐isocomene were less abundant in chemotype 3 than in chemotypes 1 and 2. Further qualitative differences were also apparent. For example, β‐santalene was present in chemotypes 1 and 2 but absent from chemotype 3, while amorpha‐4,11‐diene was present only in chemotype 3. Together, these patterns suggest that tansy roots share a sesquiterpenoid‐dominated profile, yet individual chemotypes differ in the relative abundance and presence or absence of several sesquiterpenoids.

### Stored root sesquiterpenoids are primarily affected by belowground herbivories

**Fig. 2:**
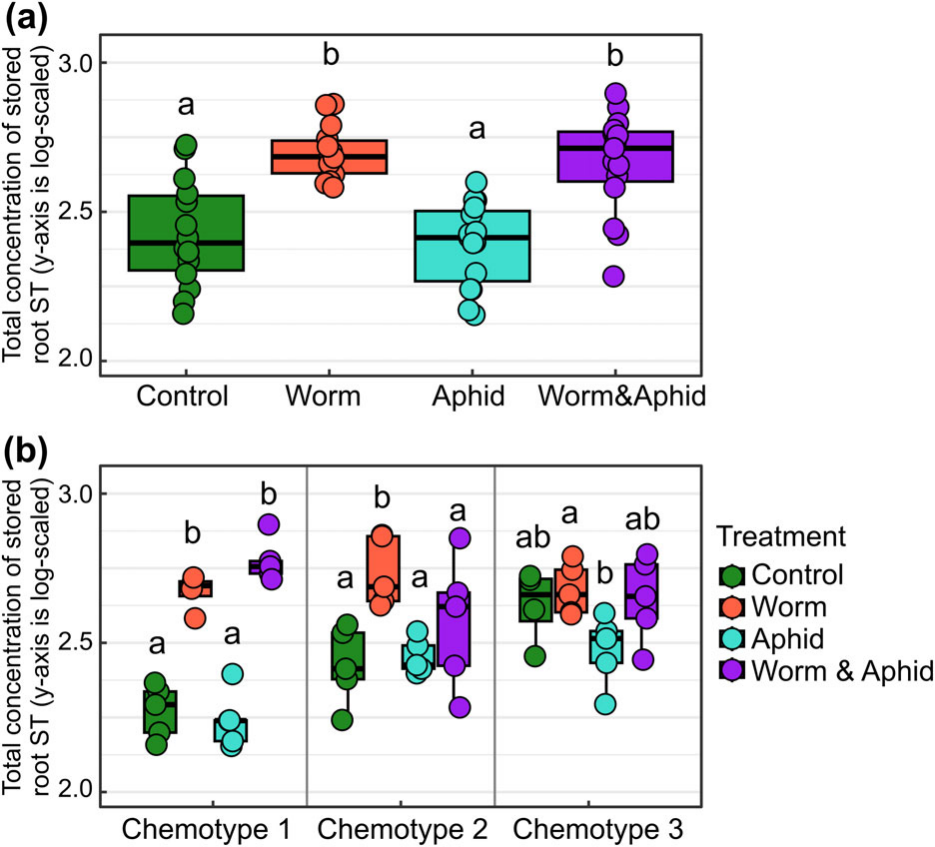
(a) Total concentration of stored root sesquiterpenoids (ST) under Control (n = 14), Worm (n = 14), Aphid (n = 15) and Worm&Aphid (n = 15) treatments. Different letters indicate significant differences among treatments (Tukey‐adjusted emmeans, α = 0.05; contrasts averaged over chemotypes). Treatments sharing different letter are significantly different. (b) Chemotypic effect of stored root STs to above and belowground feeding herbivories. Sample size per treatment were: Chemotype 1 – Control 5, Worm 4, Aphid 5, Worm&Aphid 5; Chemotype 2 – Control 5, Worm 5, Aphid 5, Worm&Aphid 5; Chemotype 3 – Control 4, Worm 5, Aphid 5, Worm&Aphid 5. Different letters indicate significant differences among treatments within each chemotype (Tukey‐adjusted emmeans, α = 0.05). All pairwise contrasts were obtained from the full model via *emmeans* (Tukey‐adjustment for multiple comparisons). The y‐axis is on a logarithmic scale. The boxes enclose the middle half of the data, with the lower edge at the 25th percentile and upper edge at the 75th percentile. The horizontal line inside each box is the median. The whiskers stretch to the most distant points within 1.5 × the box's height (the interquartile range).

Belowground feeding by wireworms strongly increased the total concentration of stored root sesquiterpenoids (F = 75.25, *P* < 0.001; Table 1 and Fig. 2a) and stored root monoterpenoids (F = 14.64, *P* < 0.001; Table 1 and Fig. S2). Plant chemotype showed a significant main effect (F = 8.91, *P* < 0.001) on root sesquiterpenoids, and the chemotypic variation regulated the extent of the wireworm response with a strong two‐way interaction (Chemotype × Worm: F = 11.56, *P* < 0.001), as the wireworms had stronger positive impacts in chemotypes 1 and 2 and less in chemotype 3 (Fig. 2b). In contrast, aboveground feeding by aphids alone did not significantly affect stored root sesquiterpenoids, and the aphid and wireworm interaction was non‐significant (Table 1). However, a significant three‐way interaction of Chemotype × Aphid × Worm (F = 4.16, *P* = 0.022) and a marginal two‐way interaction between aphid and chemotype (F = 3.01, *P* = 0.060) showed that the combined effect of wireworm and aphid on root sesquiterpenoids is likely chemotype‐dependent. This pattern is indicated by the differences between wireworm and aphid impacts relative to control and aphid impacts across chemotypes, which was strongly increased in chemotype 1, but not affected in chemotype 2–3 (Fig. 2b). We also tested the effect of naturally occurring biotic variables on the response variables. Powdery mildew was significant on stored root sesquiterpenoids (F = 5.84, *P* = 0.020; Fig. S3), while thrips and *C. tanacetina* showed no significant effects (Table 1).

**Table 1:** Analysis‐of‐variance summary for the linear model.

chemotype and biotic variables	df	stored compounds				emitted compounds	
		root MT (F, *P*‐values)	root STs (F, *P*‐values)	shoot MTs (F, *P*‐values)	shoot STs (F, *P*‐values)	shoot MTs (F, *P*‐values)	shoot STs (F, *P*‐values)
Aphid	1	1.53 (0.230)	2.69 (0.109)	1.30 (0.261)	5.17 (0.028)*	6.51 (0.014)*	9.31 (0.006)**
Chemotype (C)	2	–	8.91 (<0.001)***	1.20 (0.312)	*2.93* (*0.064*)	9.35 (<0.001)***	5.38 (0.012)*
Worm	1	14.64 (0.001)***	75.25 (<0.001)***	0.14 (0.714)	0.00 (0.949)	0.15 (0.698)	0.44 (0.513)
Aphid:C	2	–	*3.01* (*0.060*)	*3.18* (*0.051*)	*3.15* (*0.053*)	3.89 (0.028)*	7.45 (0.003)**
Aphid:Worm	1	0.62 (0.440)	0.06 (0.802)	0.00 (0.946)	0.01 (0.906)	0.16 (0.693)	1.02 (0.324)
C:Worm	2	–	11.56 (<0.001)***	0.49 (0.618)	0.62 (0.543)	0.52 (0.599)	0.80 (0.460)
C:Aphid:Worm	2	–	4.16 (0.022)*	0.01 (0.992)	0.02 (0.980)	1.43 (0.250)	0.71 (0.504)
Thrips	1	0.54 (0.471)	1.82 (0.185)	0.89 (0.350)	0.09 (0.767)	–	–
Powdery mildew	1	0.64 (0.433)	5.84 (0.020)*	1.36 (0.250)	0.55 (0.460)	–	–
*Coloradoa t*.	1	0.10 (0.758)	0.29 (0.590)	0.07 (0.797)	1.08 (0.303)	–	–

The model depicts the effects of wireworm (Worm; *Agriotes* spp.), aphid (*Macrosiphoniella tanacetaria*), chemotype and unplanned biotic variables (thrips, powdery mildew, *Coloradoa tanacetina*) on stored (root and shoot) and emitted (shoot headspace) terpenoids. Response variables are stored root monoterpenoids (MTs) and sesquiterpenoids (STs), stored shoot MTs and STs, and emitted shoot MTs and STs. Degrees of freedom (Df) and the F statistics with their associated *P*‐values are listed in the table. Significant values (*P* < 0.05) are highlighted in bold, and marginally significant values (0.05 < *P* < 0.1) in italics, and significance levels are indicated as * *P* < 0.05, ** *P* < 0.01, and *** *P* < 0.001. *P*‐values smaller than 0.001 are reported as ‘<0.001’. In the stored root MT analysis, we omitted the chemotype factor because excluding zero responses for the log transform left some chemotype * treatment combinations empty, making several coefficients aliased (non‐estimable). For the emission models, thrips, powdery mildew and *C. tanacetina* were excluded because headspace sampling occurred 10 days before the final harvest, whereas these covariates were assessed at harvest and thus including them would confound timing. Residual df by model: stored root MT = 21; stored root ST = 43; stored shoot MT = 44; stored shoot ST = 44; emitted shoot MT = 45; emitted shoot ST = 23.

We detected a significant aphid‐treatment effect on the total concentration of stored shoot sesquiterpenoids (F = 5.17, *P* = 0.028), with ~24% lower sesquiterpenoid concentrations under aphid treatment than in controls. In contrast, stored shoot monoterpenoids were not significantly affected by any biotic variable, chemotype or their interaction terms. Except for the Aphid × Chemotype interaction which was borderline for both monoterpenoids (F = 3.18, *P* = 0.051) and sesquiterpenoids (F = 3.15, *P* = 0.053). Wireworm showed no main effects, and no interactions involving wireworm were significant for either terpenoid class in shoots.

### Chemotype‐specific responses of root sesquiterpenoid *building blocks*

**Fig. 3:**
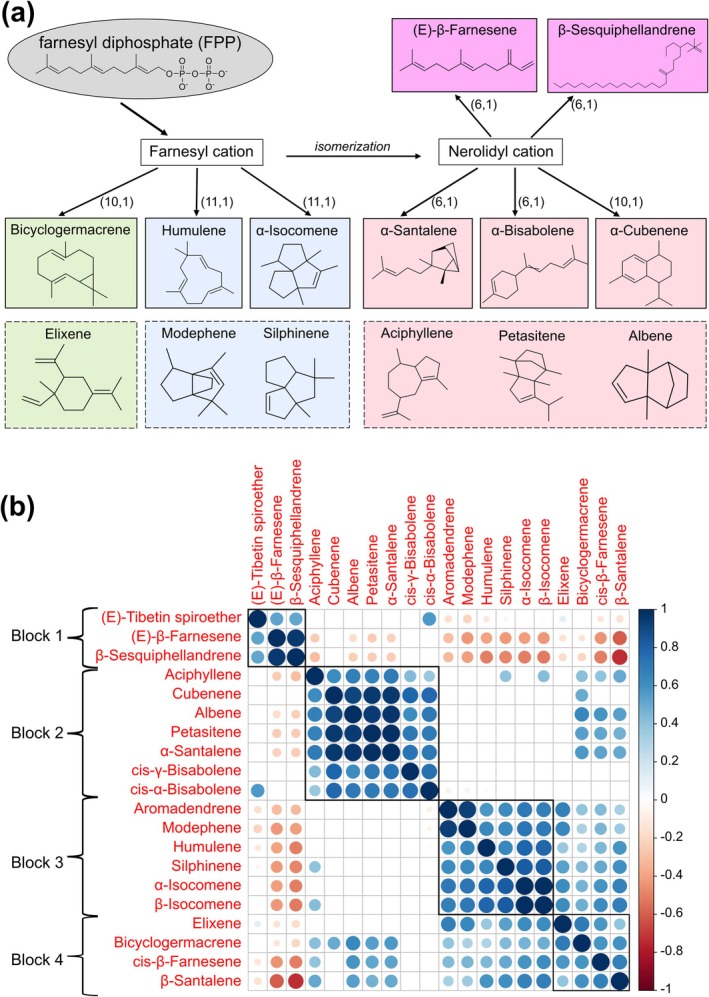
(a) Tansy root sesquiterpenoid classification scheme from the parent cations produced from farnesyl diphosphate. The numbers on top of each arrow show the cyclization from each cation. Four different colours show the corresponding sesquiterpenoids from different building blocks. The compounds highlighted with the dashed lines are proposed to show a biosynthetic relationship with the compound from the same pathway based on a strong correlation between them. The scheme was drawn using ChemDraw (version 23.1.2). (b) The correlogram showed pairwise associations among the stored root sesquiterpenoids clustered in four building blocks, indicating a likely linked biosynthesis. Circle colours show the direction of the correlation: blue – positive, red – negative. Cells left blank correspond to non‐significant correlations (*P* > 0.05). Black squares labelled Block 1 – Block 4 are the building blocks identified using hierarchical clustering. All samples (n = 58) were used in the correlation analysis.

**Fig. 4:**
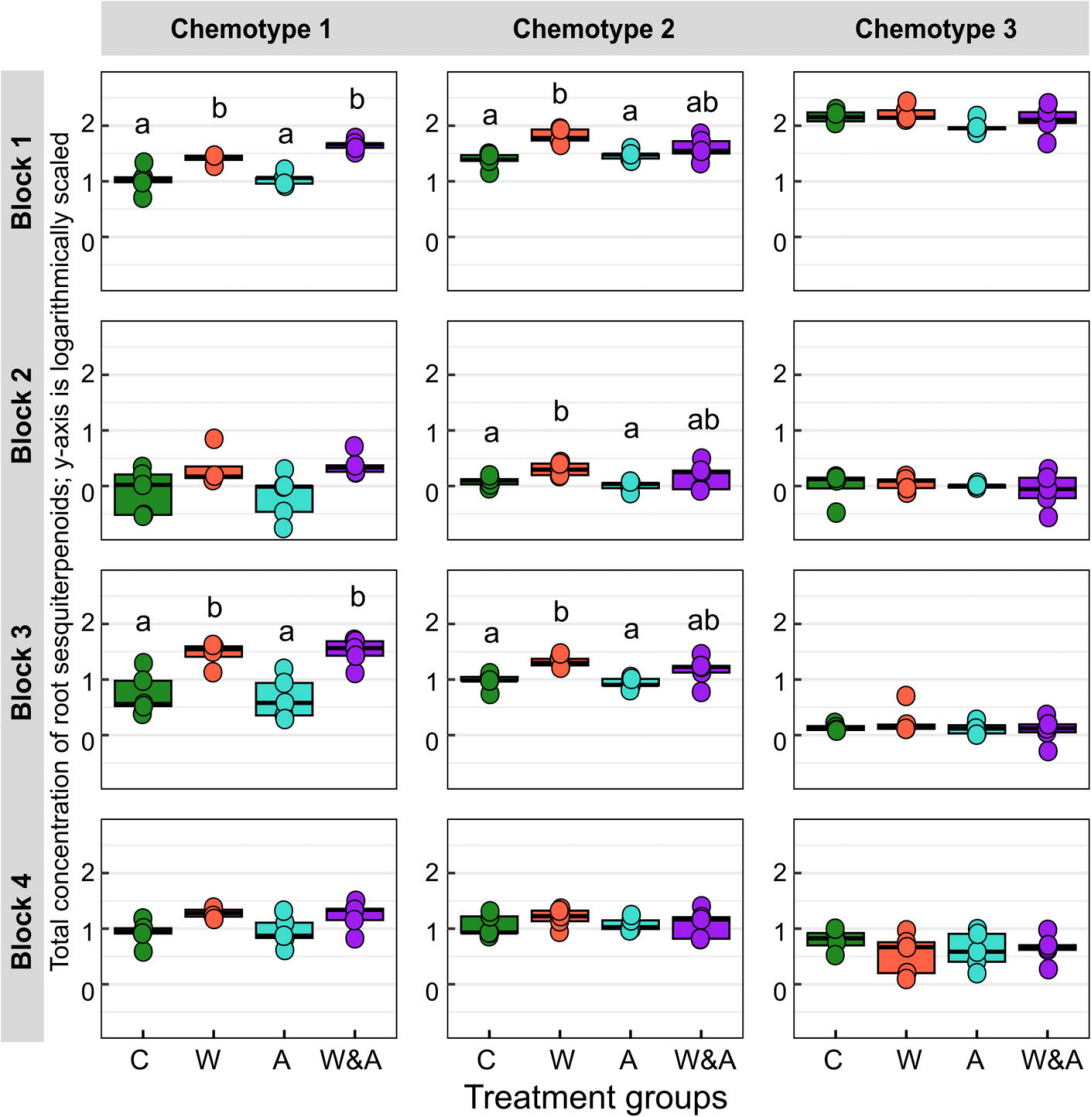
Block‐wise response of stored root sesquiterpenoids to herbivore treatments across chemotypes. Blocks are defined based on the clustering of the root sesquiterpenoids shown in Fig. 2b. Panels are arranged by block (rows 1–4) and chemotype (columns 1–3). Boxes show the interquartile range with medians; points are individual plants. Treatments on the x‐axis: C, Control (green); W, Wireworm (red); A, Aphid (cyan); W&A, Wireworm & Aphid (purple). Within each panel, treatments were compared by one‐way anova (factor Treatment) followed by Tukey *post hoc* tests. Letters show Tukey groupings: same letter = not significant (adjusted *P* ≥ 0.05); different letters = significant (adjusted *P* < 0.05). Panels without letters had a non‐significant test and thus no pairwise groupings are shown. Sample sizes were: chemotype 1 (Control 5, Worm 4, Aphid 5, Worm&Aphid 5); chemotype 2 (Control 5, Worm 5, Aphid 5, Worm&Aphid 5); chemotype 3 (Control 4, Worm 5, Aphid 5, Worm&Aphid 5).

Pairwise correlations calculated for the 20 stored root sesquiterpenoids revealed strong associations between them (Fig. 3b). Hierarchical clustering (Ward's method) outlined four highly related groups, hereafter called building blocks, that are likely the products of farnesyl or nerolidyl cations in tansy (Fig. 3a). Within each block, most pairwise correlation coefficient values exceeded 0.7 (blue circles), supporting the proposal that each block represents a distinct set of co‐varying sesquiterpenoids (Fig. S4) that likely share a common biosynthetic basis. Compounds highlighted by dashed frames in Fig. 3a (*e.g*., modephene and petasitene) showed the strongest positive links to their proposed ‘parent’ product, supporting the biosynthetic relationship. We used Sesquiterpenoid Synthase Database (bioinformatics.nl, Wageningen University & Research, n.d. see the full link in the references), an annotated survey of more than 300 plant sesquiterpenoids to show whether products originate from a farnesyl or nerolidyl cation. Block‐wise responses of sesquiterpenoids to herbivores also strongly differed among chemotypes (Fig. 4). In chemotype 1, wireworms consistently doubled the concentration of Block 1 (dominated by β‐sesquiphellandrene) and Block 3 (dominated by α‐ and β‐isocomene), and in Block 1, the dual attack enhanced the increase even further relative to control and aphid treatment. In chemotype 2, blocks 1, 2 and 3 showed a modest rise in wireworm groups, while in chemotype 3, none of the blocks responded to any herbivore treatment, mirroring its generally muted sesquiterpenoids plasticity.

### Volatile terpenoids of tansy showed minor shifts in shoots but not in roots

**Fig. 5:**
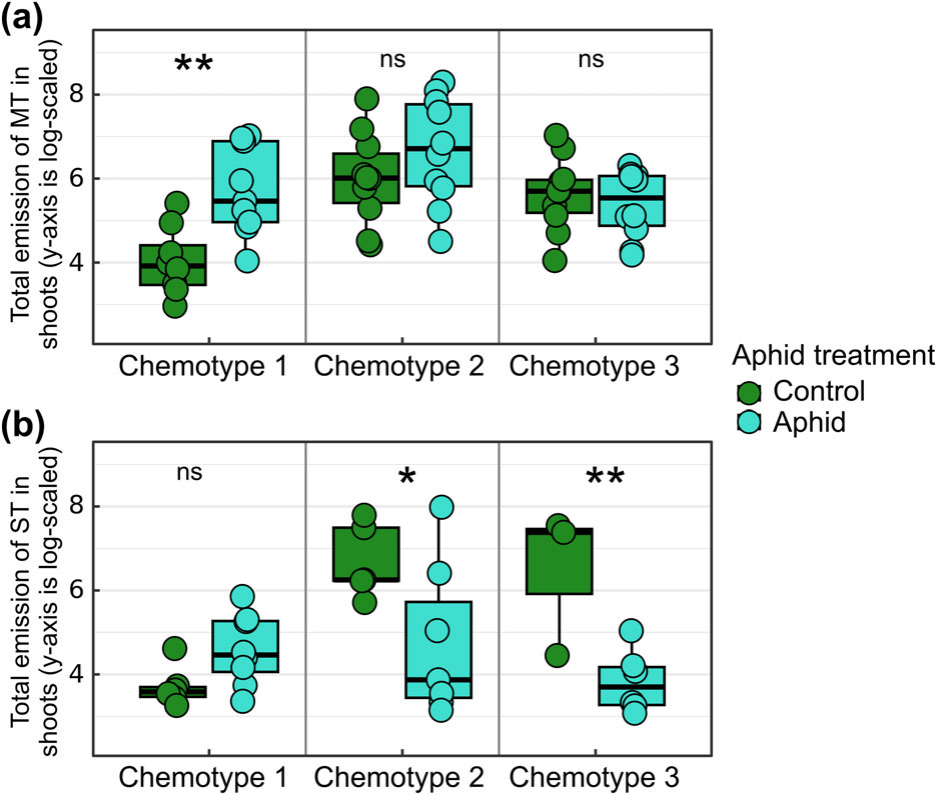
Boxplots of (a) total monoterpenoid (MT) and (b) total sesquiterpenoid (ST) emissions (headspace analysis) from shoots under Control *versus* Aphid treatments. Individual points show sample values and boxes span the interquartile range with horizontal median lines, and vertical grey lines separate chemotype blocks. Simple‐effect contrasts of Aphid within each chemotype were obtained from the full model using *emmeans* averaging over Worm and *P*‐values Bonferroni‐adjusted across chemotypes. Significance is indicated in the panels using standard symbols: ns shows a non‐significant difference between Control and Aphid within a chemotype (*P* ≥ 0.05), whereas asterisks indicate statistically significant differences, with thresholds of *P* < 0.05, <0.01 and <0.001 for *, ** and ***, respectively (based on the adjusted *P*‐values used for these contrasts). Sesquiterpenoid results should be interpreted with caution because ST were detected in only 35 of 58 plants. The ‘Control’ legend pools plants where aphids are absent (Control + Worm; green) and the ‘Aphid’ legend pools plants where aphids are present (Aphid + Worm&Aphid; turquoise). Positive sample counts in panel (a) were: chemotype 1 (Control 8, Aphid 9); chemotype 2 (Control 10, Aphid 10); chemotype 3 (Control 10, Aphid 10) and in panel (b): chemotype 1 (Control 6, Aphid 8); chemotype 2 (Control 5, Aphid 7); chemotype 3 (Control 3, Aphid 6).

The monoterpenoids emission in shoots strongly increased in the presence of aphids (Aphid main effect: F = 6.51, *P* = 0.014, Table 1), also varying by chemotype (Chemotype main effect: F = 9.35, *P* < 0.001). The significant interaction between aphid and chemotype (F = 3.89, *P* = 0.028) showed that the emission of monoterpenoids in shoots was higher under aphid presence in chemotype 1, but not in chemotype 2–3 (Fig. 5a). Similarly, the main effects of aphid, chemotype and their interaction term were significant on sesquiterpenoid emission (Fig. 5b). However, these statistical terms should be interpreted cautiously, provided that sesquiterpenoid were detected in only 35 of 58 plants, yielding an unbalanced replication between control and aphid treated plants. Neither the wireworm main effect nor any interaction terms involving wireworm were significant.

**Fig. 6:**
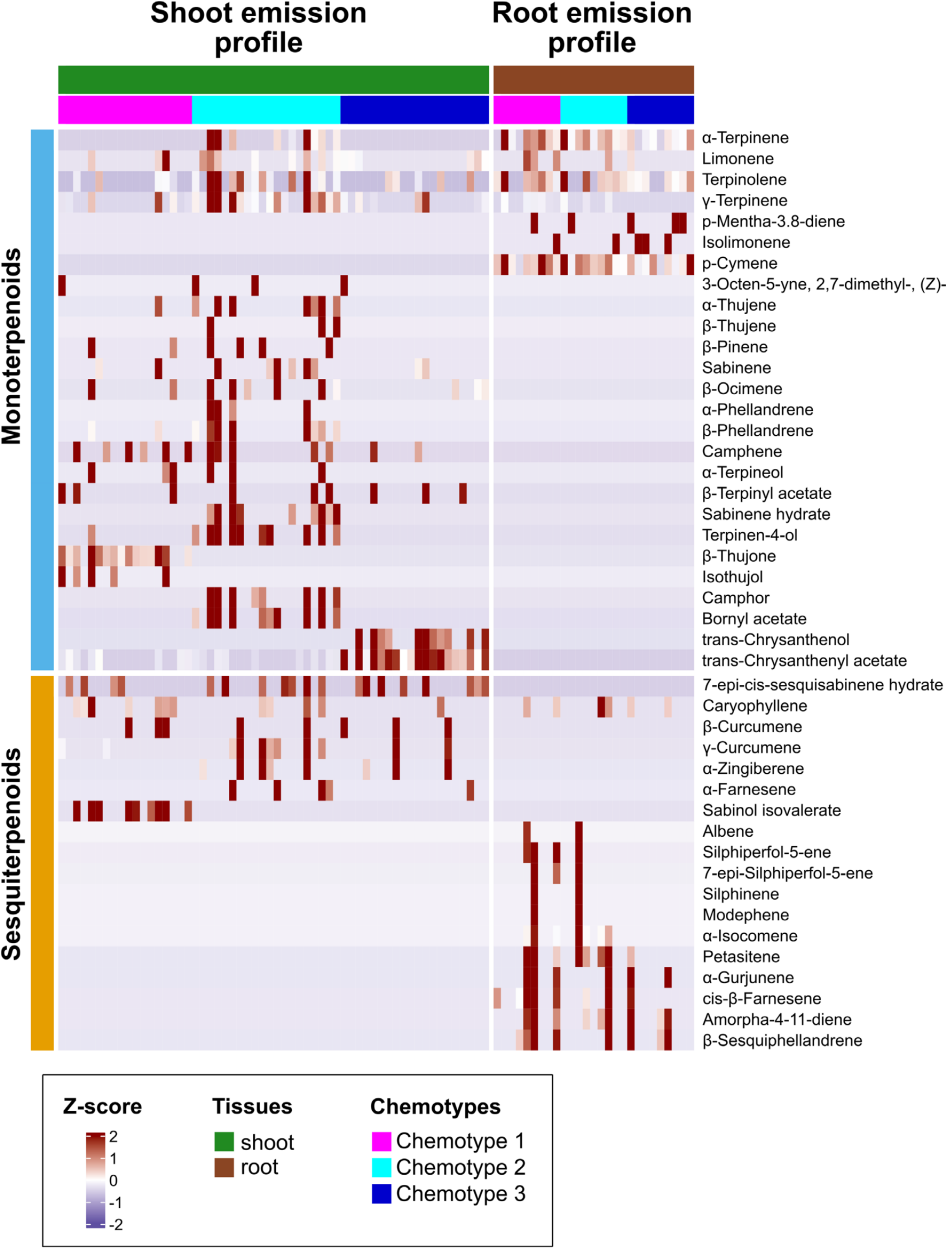
Heatmap of emitted terpenoid profiles in tansy shoot and root tissues across chemotypes, highlighting the patterns of tissue‐ and chemotype‐specific and compound‐class separation of tansy. Each column is an individual plant grouped first by shoot (in green bar) and root tissues (in brown bar) and by chemotypes: chemotype 1 in magenta, chemotype 2 in cyan and chemotype 3 in blue. Monoterpenoids are displayed by sky blue bar in the top block, and sesquiterpenoids by gold bar in the bottom block. Cells show row‐wise *Z*‐scores: for each compound, values were log‐transformed and standardized across all samples. Colours therefore represent relative abundance for that compound (red = above its mean; blue = below its mean), not biological up/down‐regulation. Because emissions are non‐negative and some entries are zero or low, many values fall below the compound mean and therefore appear pale blue to near‐white, whereas a subset of samples with higher emissions appears red. Root emissions were generally lower and less frequent than shoot emissions and consequently, root columns show predominantly low relative values for many compounds (often pale/blue). Root‐emitted terpenoids presented in this figure are from top‐zone measurement.

Root volatile emissions were generally low, but they were spatially structured within the rhizotron. The upper 3–5 cm (‘top‐zone’), where coarse roots are concentrated, emitted substantially more monoterpenoids than the lower‐root zone (Bonferroni‐adjusted Dunn tests: top‐ *versus* low‐zone *P* < 0.001; Fig. S5a). Despite a few outliers for sesquiterpenoids in the low‐zone, the top‐zone also showed higher sesquiterpenoid emission (*P* = 0.025; Fig. S5b). Although the top‐zone sampler clearly captured root‐emitted mono‐ and sesquiterpenoids, the number of positive samples was limited (root‐emitted mono‐: 25; root‐emitted sesquiterpenoids: 13). Because root VOC emissions were detected (due to very low abundance) in only a subset of samples and sampling was zone‐specific, we did not include root VOCs in the multivariable model summarized in Table 1. Accordingly, we present these root emissions descriptively and reserve formal hypothesis testing for a follow‐up experiment with greater replication. Additionally, we visually examined tissue‐ and chemotype‐specific patterns in emitted terpenoids on a heatmap. Samples clustered primarily by tissue, clearly separating shoots from roots (Fig. 6), consistent with expected differences between biosynthetic classes. Shoot emissions were dominated by monoterpenoids: 22 monoterpenoids were detected in shoots, whereas roots emitted only seven, four of which (α‐terpinene, limonene, terpinolene, γ‐terpinene) also occurred in shoots. By contrast, sesquiterpenoids were scarce in shoots but abundant and diverse in roots. Within each tissue, the emission bouquets varied with chemotype, broadly echoing the foliar chemotype classification seen in stored profiles.

### Chemotypes differ in root‐system development and allocation dynamics

**Fig. 7:**
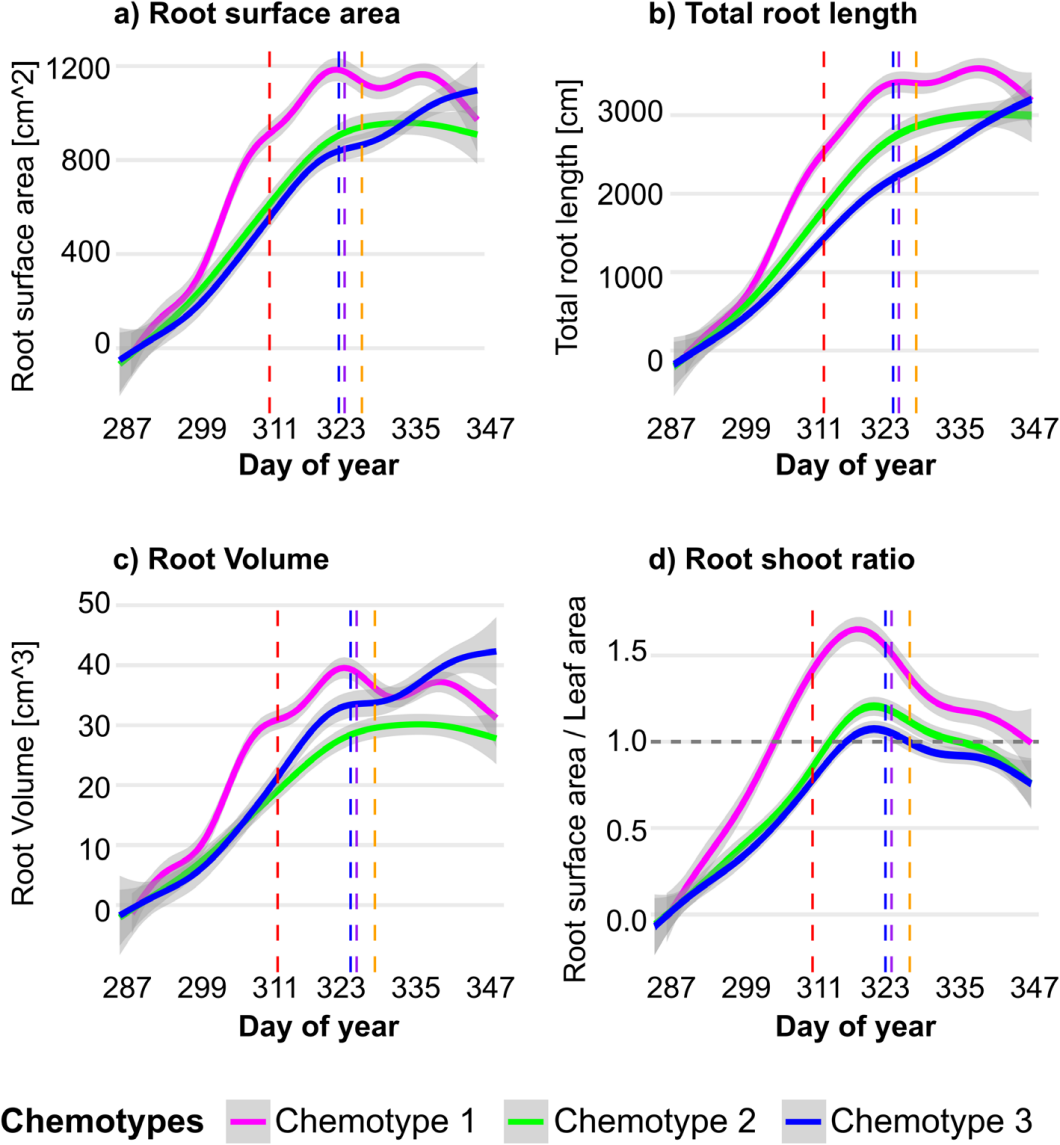
Growth dynamics of root traits by chemotypes. Curves are generalized additive model (GAM) smooths (thin‐plate splines, *k* = 10; restricted maximum likelihood (REML)) fitted separately by chemotype, with 95% confidence ribbons. Panels show (a) root surface area, (b) total root length, (c) root volume and (d) root:shoot ratio over the days of year (day 287–347). Magenta, green and blue lines correspond to chemotype 1, 2 and 3, respectively. The vertical dashed lines depict different events that occurred during the experiment; from left to right: the start of wireworm treatment (day 313) and of aphid treatment (day 325), twister analysis in the rhizotrons (day 326), initial harvesting (day 329), respectively. The initial harvesting was conducted for transcriptomic analysis, which is not part of the current study. Chemotype 1 exhibits the most rapid early root expansion and reaches its maximum root:shoot ratio around day 320.

Time‐course trajectories (Fig. 7) demonstrated that root traits varied systematically among chemotypes throughout the monitoring period. Across all four metrics – root surface area, total root length, root volume and root‐to‐shoot ratio (in terms of root surface area / leaf area), chemotype 1 consistently produced the largest values, followed by chemotype 2 and 3 until around day 335 (12 days before the end of the experiment). Between days 320 and 330 (almost 9 weeks after the start of the experiment), chemotype 1 roots covered the highest values across traits (Fig. 7). Over this interval, chemotype 2 reached about 80%–90% and chemotype 3 about 70%–80% of chemotype 1. All chemotypes followed a sigmoidal pattern but differed in growth. Chemotype 1 displayed the steepest rise until around day 330, and later reached its inflection point a few days earlier than chemotype 3 for surface area and length. Root volume, however, showed a substantial drop in chemotype 1 from around day 335, whereas volume in chemotype 3 maintained continuous growth until the end of the experiment. Chemotype 2 entered the saturation phase around the same time as chemotype 1. The reason chemotype 3 eventually exceeds the others in volume is because of its larger median root diameter (Fig. S6), showing that this chemotype produced fewer but thicker roots. The root:shoot surface ratio peaked at about 1.7 in chemotype 1, 1.3 in chemotype 2 and 1.1 in chemotype 3 before all three converged toward ~1.0 as leaf canopies expanded. Pairwise contrasts (Table S1) showed that chemotype 1 had higher values than the others for root surface area, total root length and root:shoot ratio (χ^2^, BH‐adjusted *P* < 0.05). For root volume, chemotype 1 exceeded chemotype 2 (*P* = 0.027), while the contrast with chemotype 3 was not significant after adjustment (*P* = 0.055). Chemotypes 2 and 3 did not differ significantly for any trait (all *P* > 0.17). Furthermore, we observed a positive (R = 0.67, *P* = 0.01) correlation (Fig. S7) between root surface area and the total concentration of stored root sesquiterpenoids within the pooled set of individual control plants that hinted at a synergetic relationship in the growth‐defence system. The evidence is, however, insufficient as our sample size is too small to claim a definitive evolutionary synergy theory.

## DISCUSSION

In this study, we demonstrate that above‐ and belowground herbivory in *T. vulgare* elicits compartment‐specific responses that reveal localized and specific defence strategies. Feeding by a belowground root herbivore‐induced production of sesquiterpenoids in the roots, whereas feeding by an aboveground phloem‐feeding herbivore‐induced predominantly volatile monoterpenoid emissions. Using different chemotype lineages, we reveal that there are both generalizable and chemotype‐specific responses to herbivory. We identify strong biosynthetic clustering highlighting terpenoid biosynthesis pathways, which are distinctly impacted by herbivory. Our findings provide strong evidence for the specific ecological roles of mono‐ and sesquiterpenoids and associated biosynthetic machinery.

Belowground wireworm feeding elevated the total concentration of stored root terpenoids while having no influence on shoot pools or emissions. Additionally, our results showed that aphids alone did not affect root terpenoids. Yet, in combination with wireworms, aphids amplified the sesquiterpenoid increase in chemotype 1 and had no detectable effect in chemotype 2 and 3. Thus, our study suggests that phloem feeders do not elicit systemic root induction on their own but can modify root defences triggered by belowground herbivores in a chemotype‐dependent manner. A comparable pattern was reported by Bezemer *et al*. (2004) who demonstrated that terpenoid‐aldehydes increased markedly in roots of *G. herbaceum* seedlings after wireworm (*A. lineatus*) feeding. While the authors demonstrated a slight systemic signal reaching the shoots, the quantitatively stronger reaction was contained in the roots. This is not at odds with the broader above‐ and belowground literature. Generally, systemic outcomes depend strongly on the inducing guild and hormone crosstalk. For instance, phloem feeders typically trigger salicylic acid (SA) and can antagonize jasmonic acid (JA) signalling (Erb & Reymond 2019), while the biosynthesis of many sesquiterpenoids is regulated by jasmonate signalling (Tholl 2015). Within this framework, the lack of general aphid‐induced responses in root sesquiterpenoids aligns with SA‐JA crosstalk. In a related study, Karssemeijer *et al*. (2020) showed that phloem‐feeding cabbage aphids (*Brevicoryne brassicae* L.) did not activate the root JA pathway, whereas chewing caterpillars (*Plutella xylostella* L.) did and this translated into reduced performance of the root herbivore *Delia radicum* L. The authors interpret this as guild‐specific systemic crosstalk (SA‐biased aphids *versus* JA‐biased chewers), consistent with our lack of JA‐dependent root sesquiterpenoid induction under aphid feeding. By contrast, systemic shoot‐to‐root responses can occur in other systems. For instance, in Arabidopsis, aboveground aphid feeding (*B. brassicae*) triggered systemic transcriptomic changes in roots, whereas root attack by *Heterodera schachtii* A. Schmidt did not produce systemic changes in shoots (Kutyniok *et al*. 2014). The authors interpret these patterns as attacker‐specific, signalling‐mediated responses under low infestation, consistent with SA‐JA crosstalk. In their earlier metabolomics work, they described ‘minute’ systemic metabolic shifts under similar conditions (Kutyniok & Müller 2012). Taken together, our data suggest that aphid feeding alone did not increase stored root sesquiterpenoid levels, but aphids modified root sesquiterpenoid responses during cross‐guild attack in a chemotype‐specific manner.

Furthermore, our findings demonstrated a pronounced chemotype‐specific induction of root sesquiterpenoids following a wireworm attack. Chemotypes 1 and 2 induced their sesquiterpenoid pools under wireworms, and the combined effect of wireworm and aphid further boosted the induction in the root chemotype 1. In contrast, chemotype 3 remained mostly unchanged. This highly chemotype‐specific response is very likely linked to building blocks of sesquiterpenoids that we identified using a hierarchical clustering method. These blocks are proposed to reflect a biochemical pattern that can arise when a single multiproduct sesquiterpene synthase catalyses the conversion of farnesyl diphosphate into multiple sesquiterpenoid products. Upon evaluating the response of each proposed block to each treatment within each chemotype, we found a significant induction effect of Block 1 (defined by β‐sesquiphellandrene) and Block 3 (defined by α‐ and β‐isocomene) triggered by wireworms in chemotype 1, thereby supporting the remarkable plasticity of this chemotype that we observed before. This block‐level and chemotype‐specific induction of root sesquiterpenoids may be linked to discrete suites of compounds that could be co‐produced by one or more multiproduct sesquiterpene synthases. For example, in spotted knapweed (*C. stoebe*), transcriptome‐guided characterization identified two root‐expressed sesquiterpene synthases (CsTPS4 and CsTPS5), and enzyme assays showed that these enzymes together can generate most of the root sesquiterpene blend (Gfeller *et al*. 2019). Similarly, in maize, the inducible β‐caryophyllene synthase TPS23 is expressed in ancestral and European lines but is transcriptionally absent in the majority of North American types, along with the entire inducible volatile module (Köllner *et al*. 2008). By analogy, the strong induction of Blocks 1 and 3 in chemotype 1 suggests that this chemotype possesses active alleles of the TPS genes for those blocks, whereas the other chemotypes probably carry silenced or low‐expression variants. Shoot transcriptomics in tansy (Clancy *et al*. 2020) identified numerous putative TPS transcripts, while herbivory‐associated differential expression was limited: reported responsive TPS candidates included a putative (−)‐germacrene D synthase (*TanvuEGr017925*) and two (E)‐β‐farnesene‐like TPSs (*TanvuEGr029614*, *TanvuEGr007220*). *TanvuEGr029614* transcripts were strongly induced by aphid feeding, whereas *TanvuEGr007220* transcript abundance increased under combined aphid and caterpillar attack. These shoot transcriptomic patterns support inducible, chemotype‐specific regulation of *TPS* genes that may contribute to variation in volatile emissions. Because β‐sesquiphellandrene and isocomene dominate tansy roots, analogous root TPSs likely exist, but remain uncharacterized. As these blocks were defined using statistical patterns alone, the next important step would be to validate the underlying biosynthetic basis by combining root transcriptomics with the functional characterization of candidate tansy *TPS* genes via their expression in a suitable model system (*e.g*., yeast or *Nicotiana benthamiana* Domin).

Following 14 days of aphid feeding and 36 h of volatile collection using twisters, headspace analysis of shoot samples indicated a significant increase in shoot monoterpenoid emissions in chemotype 1 (β‐thujone dominated in shoots), while chemotype 2 (camphor and sabinene‐hydrate dominated) and 3 (trans‐chrysanthenyl‐acetate dominated) exhibited no variation, suggesting that the emission of monoterpenoids under aphid attack is specific to chemotypes. Solvent extracts of the same shoots demonstrated only weak alteration in stored monoterpenoid pools, a pattern that aligns with the kinetic model in which sap‐feeding insects can upregulate *TPS* transcript levels, resulting in a rapid *de novo* ‘emission pool’ while the slow turnover reservoirs in glandular trichomes remain mostly unaffected (Niinemets *et al*. 2013; Clancy *et al*. 2020). Why is the emission of monoterpenoids favoured? Monoterpenoids generally have higher vapour pressures than sesquiterpenoids, allowing them to diffuse out easily through the cuticle, whereas sesquiterpenoids, having higher boiling points and lower vapour pressures, are often retained in storage pools and are emitted slowly from intact leaves (Mofikoya *et al*. 2019). These physicochemical differences help explain why aphid feeding significantly enhanced monoterpenoid emissions compared to sesquiterpenoids, given the importance of enzymatic regulation in *de novo* synthesis (Niinemets *et al*. 2013). Clancy *et al*. (2020) likewise documented analogous storage *versus* emission decoupling and further revealed that aphid pre‐infestation amplified the caterpillar‐induced monoterpenoid spike in certain tansy chemotypes, but not in others. Our findings indicate that phloem‐feeding aphids induce a chemotype‐specific, *de novo* release of readily diffusive terpenoids, while primarily preserving the storage pools. We additionally collected the terpenoids from roots and found that the tissue‐specific partitioning of emitted terpenoids parallels our earlier findings on stored pools (Rahimova *et al*. 2025), where mono and sesquiterpenoid profiles were clearly localized between shoots and roots. Despite a substantial number of replications, mono‐ and sesquiterpenoids were detected in only a few root samples, limiting our ability to statistically assess herbivory impacts. To effectively evaluate whether wireworms alter root terpenoid emissions, a specific, root‐emission‐focused methodology is required. For instance, Lee Díaz *et al*. (2022) used a sterile two‐compartment Petri dish and passively sampled root VOCs in situ from tomato seedlings 24 h after *Spodoptera exigua* Hübner attack, using HiSorb/PDMS sorbents and TD‐GC–QTOF–MS. In addition to demonstrating an in situ capture setup, they indicated that root volatiles vary under herbivory and that the choice of methodology (HiSorb *versus* PDMS) influences the observed outcomes, advocating for multi‐sorbent sampling.

Our results showed that chemotype identity is linked to not only terpenoid chemistry but also to root architecture. Chemotype 1 maintained the largest root surface area and length and the highest root:shoot ratio across the pre‐harvest window. Chemotype 2 followed a similar rise but levelled off earlier at lower values. Chemotype 3 had the smallest surface area and length yet formed comparatively thicker roots (higher median diameter) and thus produced the larger root volume toward the end of the experiment. Because in tansy, coarse roots contain a higher total sesquiterpenoid concentration than fine roots (Rahimova *et al*. 2025), a greater fraction of coarse roots in chemotype 3 provides the plausible explanation for its higher constitutive root sesquiterpenoid storage that we observed in the present study (Fig. 1, control boxes). Additionally, a positive linkage between total root sesquiterpenoids and surface area suggests a beneficial association between root‐growth capacity and inducible defence, rather than a classic growth‐defence trade‐off. A comparable ‘*defence‐supports‐growth*’ effect has been documented in the *Populus–Laccaria* symbiosis, where young poplar trees associate with the ectomycorrhizal fungus *Laccaria bicolor* (Maire) P.D. Orton. The fungus releases a bouquet of volatile sesquiterpenoids dominated by (−)‐thujopsene that acts as an airborne signal to the plant. Exposure to this sesquiterpenoid blend reprogrammed the poplar's root system, increasing the number and length of lateral roots and greatly expanding root surface area (Ditengou *et al*. 2015). Together with prior studies, our results suggest that chemotype co‐varies with morphological traits and observed ‘chemotype effects’ could stem from terpenoid chemistry itself or from other co‐varying traits. Distinguishing these alternatives will require targeted causal tests to investigate this putative growth and sesquiterpenoid induced defence theory.

## CONCLUSIONS

In conclusion, the present study demonstrated that tansy exhibits compartmentalized, chemotype‐dependent defence responses, with induction differing between roots *versus* shoots and between stored pools *versus* volatile emissions. Wireworm feeding triggers a response in the mono and sesquiterpenoid concentrations in the roots yet fails to deliver any measurable upward signal to the shoots. Whereas aphid attack on shoots elicits a *de novo* burst of monoterpenoid emissions without altering root chemistry. Direct quantification of JA and SA would be an important future step to test the hormonal basis of these responses. Among the three chemotypes, chemotype 1 launched the strongest inducible defence both in shoot and root tissues and produced larger root systems and higher root:shoot ratio. Collectively, these patterns indicate that tansy allocates its inducible chemistry and root architecture among tissues in a manner that depends on both the attacker and the plant's identity. The subsequent crucial phase is to conduct a high‐quality *T. vulgare* transcriptomic analysis. Such a platform will allow us to identify terpenoid synthase (TPS) genes that underlie each sesquiterpenoid ‘block’, and link genetic variation in TPS modules to both inducible chemistry and root system architecture.

## AUTHOR CONTRIBUTIONS

J‐PS, RH and HN designed the experiment; HN gathered and evaluated the raw data, performed the statistical models, drafted the figures and the manuscript with the guidance from J‐PS, RH and WW; GG, PK and AS set up the experiment in the greenhouse; RH, AN and LO‐P propagated the plants; HN, CMV, GG, PK, RH, AN and LO‐P performed the final 4 days of harvesting; CMV further analysed and extracted the root images and features with the supervision of JBW; HN and IZ conducted the shoot and root extraction analysis; BW performed GC–MS runs and calibration analysis; J‐PS and JBW supervised the 60 day of experiment in the greenhouse.

## Supporting information


**Fig. S1.** Treatment timeline of the tansy greenhouse experiment. Each horizontal bar represents one treatment: an unmanipulated control, wireworm only, aphid only and wireworm and aphid groups. During the initial stabilization phase of the plants, all plants were maintained under identical conditions. Green segments show the establishment period (Day of year, 285–313), orange segments the wireworm phase (started on day 313) and blue segments the aphid phase (started on day 325). The experiment ended on day 347.
**Fig. S2.** Boxplots of the log‐transformed total concentration of stored root monoterpenoids (MT) in Control *versus* Worm‐treated plants. Worm‐treated roots had higher monoterpenoids than controls (Type III anova on full model: Worm effect F = 14.62, *P* = 0.001). Significance is indicated in the panel using asterisks. Sample sizes per treatment: Control 9, Worm 19.
**Fig. S3.** Distribution of total concentration of stored root sesquiterpenoids (ST) across mildew infection levels. Boxplots show values (y‐axis on a log scale) for plants with none, mild and high mildew infection. The overall mildew effect was significant in the full model (Type III anova: F = 5.84, *P* = 0.020). Positive sample counts by infection level were: none 37, mild 11, high 10.
**Fig. S4.** Relative abundance of stored root sesquiterpenoids in each building block. Block 1 is mostly dominated by β‐Sesquiphellandrene, Block 2 by Albene, Block 3 by α‐ and β‐Isocomene, and Block 4 by cis‐β‐Farnesene.
**Fig. S5.** Root (a) monoterpenoid and (b) sesquiterpenoid emissions across rhizotron zones: (1) top‐zone – twister ~5 cm below the surface (near coarse roots); (2) low‐zone – twisters on individual lower roots (30–40 cm from the top) enclosed with foil bags. The soil‐background check showed no terpenoid peaks and was therefore omitted from the figure and analysis. Bonferroni‐adjusted Dunn tests showed higher monoterpenoid and sesquiterpenoid emission in the top‐zone relative to the low‐zone (MT: ***; *P* < 0.001; SQT: *; *P* = 0.025). Positive sample sizes were: top‐zone 25, low‐zone 36 in panel a and top‐zone 13, low‐zone 34 in panel b.
**Fig. S6.** Median root diameter over the days of year (DOY 287–347) differed by chemotypes. Magenta, green and blue curves show chemotype 1, chemotype 2 and chemotype 3. Vertical markers show, from left to right, the onset of wireworm treatment (day 313), aphid treatment (day 325), the rhizotron twister analysis (day 326) and the initial harvest (day 329).
**Fig. S7.** Root surface area (mm^2^) measured a week before the final harvest is plotted against the total concentration of stored sesquiterpenoids (Pearson's R = 0.67, *P* = 0.01) in the roots of untreated plants (n = 14). The solid line shows the fitted linear regression with the 95% confidence interval (grey band).
**Table S1.** Pairwise chemotype contrasts for root traits from generalized additive mixed models (GAMMs). Models were fit with mgcv using restricted maximum likelihood (REML) and included chemotype‐specific smooths of day of year (factor‐smooth, *k* = 10) and a plant‐level random intercept. Estimated marginal means were averaged uniformly over the observed time range. Pairwise differences were tested with Wald χ^2^ (df = 1) from the estimated marginal means (EMM) contrasts, and *P*‐values were Benjamini‐Hochberg‐adjusted within trait. We limited statistical inference to data collected between day 287 and 329 (from the first measurement until the initial harvest) as this time window provided the most complete, uninterrupted observations.

## Data Availability

All data supporting this study (GC–MS tables, retention indices/retention times, mirror EI mass spectra for identified terpenoids, imaging‐derived root traits and R scripts) have been deposited on the Open Science Framework (OSF). The registration of data can be found in OSF under the following DOI: 10.17605/OSF.IO/CG5H8.
