# Supplementary material for: Organ‐specific terpenoid responses in Tanacetum vulgare are chemotype‐dependent
Source: Plant Biol (Stuttg). 2026 Apr 1;28(5):1437–53. doi: 10.1111/plb.70208 (PMC13358675; doi:10.1111/plb.70208)
Supplement: Supplementary file 1 — Fig. S1. Treatment timeline of the tansy greenhouse experiment. Each horizontal bar represents one treatment: an unmanipulated control, wireworm only, aphid only and wireworm and aphid groups. During the initial stabilization phase of the plants, all plants were maintained under identical conditions. Green segments show the establishment period (Day of year, 285–313), orange segments the wireworm phase (started on day 313) and blue segments the aphid phase (started on day 325). The experiment ended on day 347. Fig. S2. Boxplots of the log‐transformed total concentration of stored root monoterpenoids (MT) in Control versus Worm‐treated plants. Worm‐treated roots had higher monoterpenoids than controls (Type III anova on full model: Worm effect F = 14.62, P = 0.001). Significance is indicated in the panel using asterisks. Sample sizes per treatment: Control 9, Worm 19. Fig. S3. Distribution of total concentration of stored root sesquiterpenoids (ST) across mildew infection levels. Boxplots show values (y‐axis on a log scale) for plants with none, mild and high mildew infection. The overall mildew effect was significant in the full model (Type III anova: F = 5.84, P = 0.020). Positive sample counts by infection level were: none 37, mild 11, high 10. Fig. S4. Relative abundance of stored root sesquiterpenoids in each building block. Block 1 is mostly dominated by β‐Sesquiphellandrene, Block 2 by Albene, Block 3 by α‐ and β‐Isocomene, and Block 4 by cis‐β‐Farnesene. Fig. S5. Root (a) monoterpenoid and (b) sesquiterpenoid emissions across rhizotron zones: (1) top‐zone – twister ~5 cm below the surface (near coarse roots); (2) low‐zone – twisters on individual lower roots (30–40 cm from the top) enclosed with foil bags. The soil‐background check showed no terpenoid peaks and was therefore omitted from the figure and analysis. Bonferroni‐adjusted Dunn tests showed higher monoterpenoid and sesquiterpenoid emission in the top‐zone relative to the low‐zone (MT: ***; P < 0.001; [file PLB-28-1437-s001.docx]

**Organ-specific terpenoid responses in *Tanacetum vulgare* are chemotype-dependent**

Humay Newrzella^1^, Robin Heinen^2^, Cecilia Manjón Villasante^1^, Ina Zimmer^1^, Baris Weber^1^, Peter Kary^1^, Georg Gerl^1^, Annika Neuhaus^2^, Alexandros Sigalas^1^, Lina Ojeda-Prieto^2,3,4^, Jana Barbro Winkler^1^, Wolfgang W. Weisser^2^, Jörg-Peter Schnitzler^1,5*^

^1^Research Unit Environmental Simulation, Helmholtz Munich, D-85764 Neuherberg, Germany

^2^Terrestrial Ecology Research Group, Department of Life Science Systems, School of Life Sciences, Technical University of Munich, D-85354 Freising, Germany

^3^Animal Ecology, Institute of Biochemistry and Biology, University of Potsdam

^4^Biodiversity Research/Systematic Botany Group, Institute of Biochemistry and Biology, University of Potsdam

^5^Ecosystem Physiology, Faculty of Environment and Natural Resources, University Freiburg, D-79110 Freiburg, Germany

**Supplementary Materials**

 
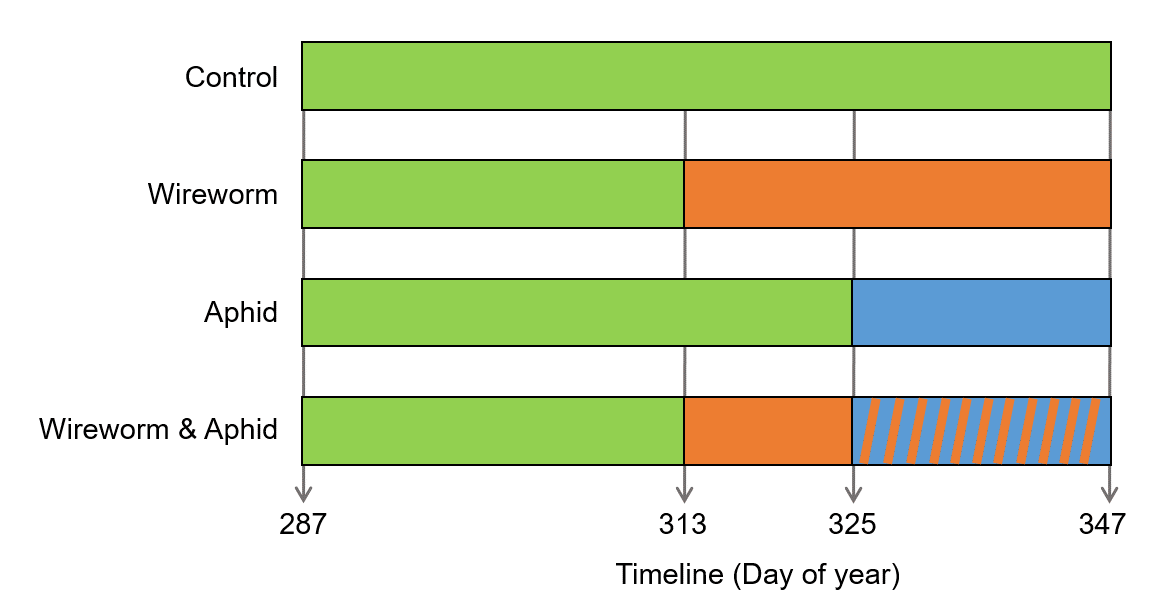


**Figure S1:** Treatment timeline of the tansy greenhouse experiment. Each horizontal bar represents one treatment: an unmanipulated control, wireworm only, aphid only, and wireworm & aphid groups. During the initial stabilization phase of the plants, all plants were maintained under identical conditions. Green segments show the establishment period (Day of year, 285–313), orange segments the wireworm phase (started on day 313), and blue segments the aphid phase (started on day 325). The experiment ended on day 347.


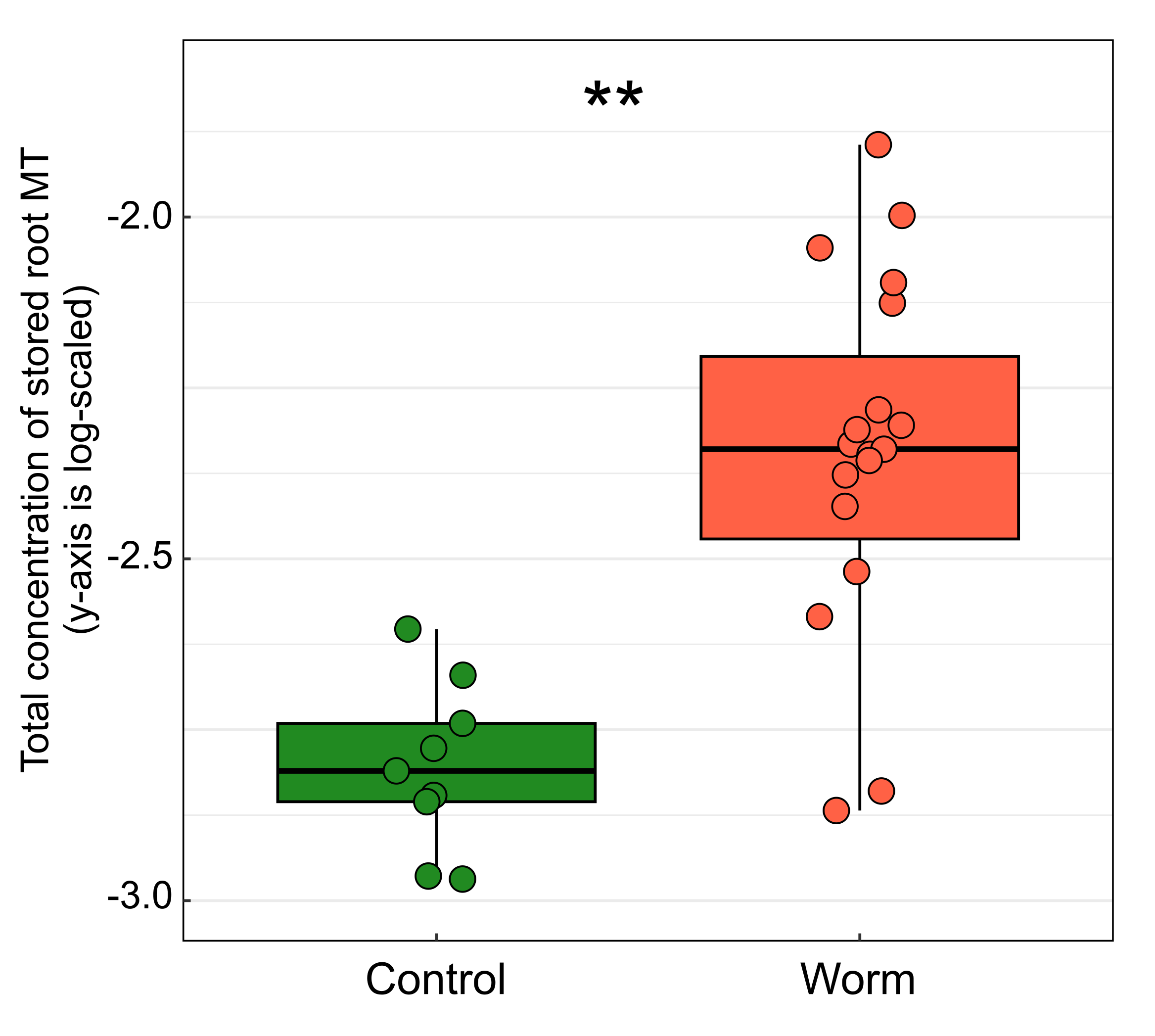


**Figure S2.** Boxplots of the log-transformed total concentration of stored root monoterpenoids (MT) in Control vs Worm-treated plants. Worm-treated roots had higher monoterpenoids than controls (Type III ANOVA on full model: Worm effect F = 14.62, p = 0.001). Significance is indicated in the panel using asterisks. Positive sample counts per treatment: Control 9, Worm 19.


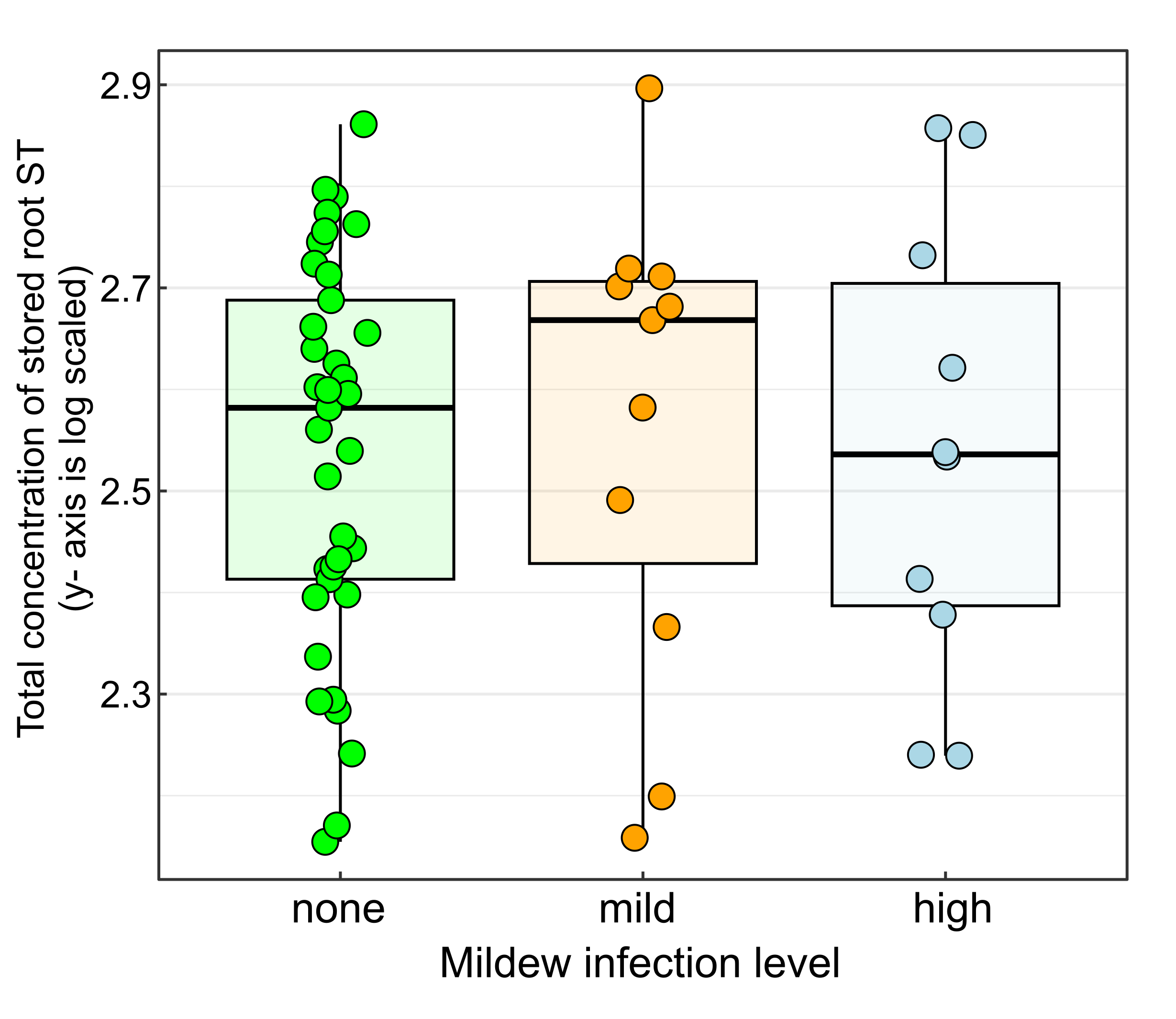


**Figure S3.** Distribution of total concentration of stored root sesquiterpenoids (ST) across mildew infection levels. Boxplots show values (y-axis on a log scale) for plants with none, mild and high mildew infection. The overall mildew effect was significant in the full model (Type III ANOVA: F = 5.84, p = 0.020). Positive sample counts by infection level were: none 37, mild 11, high 10.


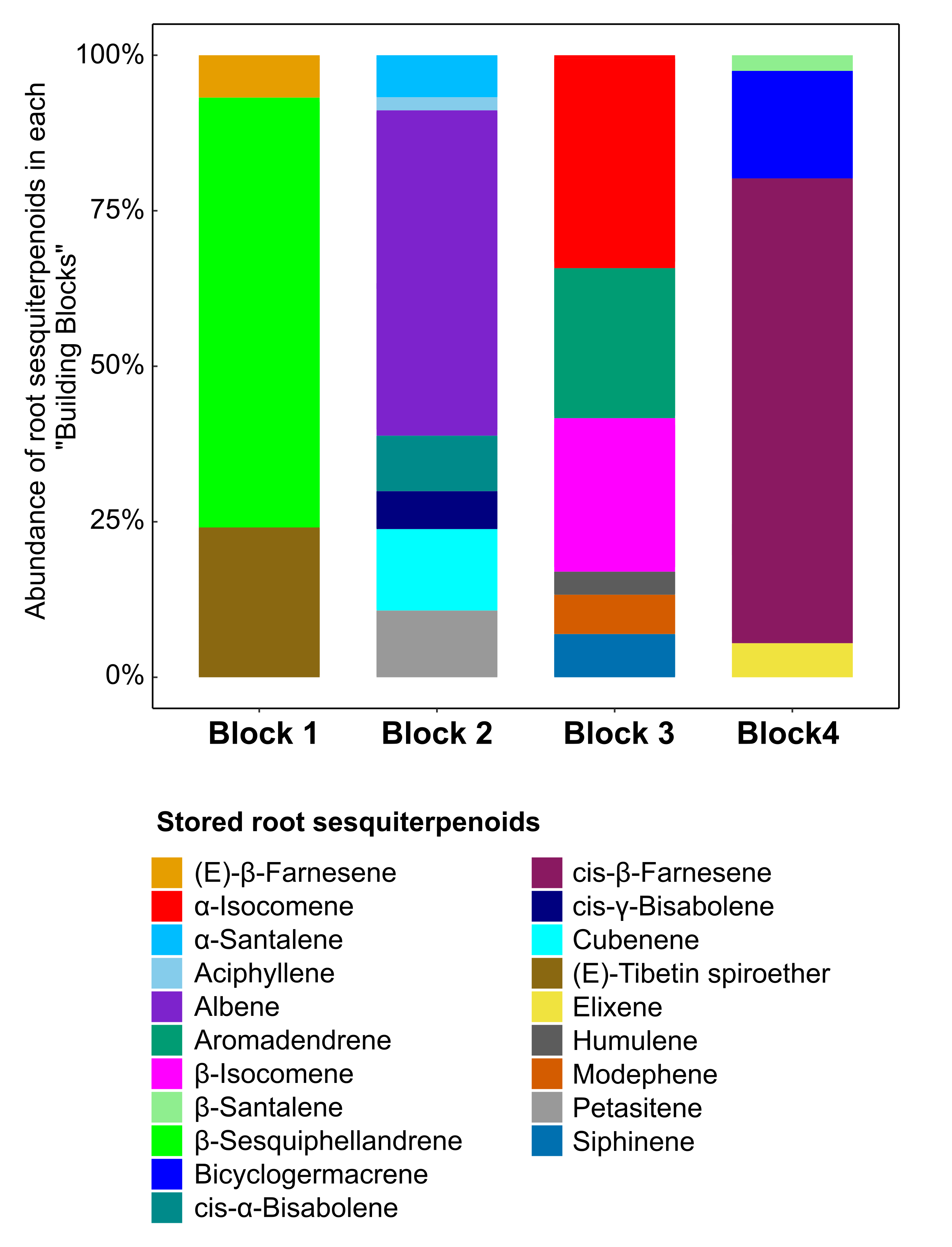


**Figure S4.** Relative abundance of stored root sesquiterpenoids in each building block. Block 1 is mostly dominated by β-Sesquiphellandrene, Block 2 by Albene, Block 3 by α- and β-Isocomene, and Block 4 by cis-β-Farnesene.


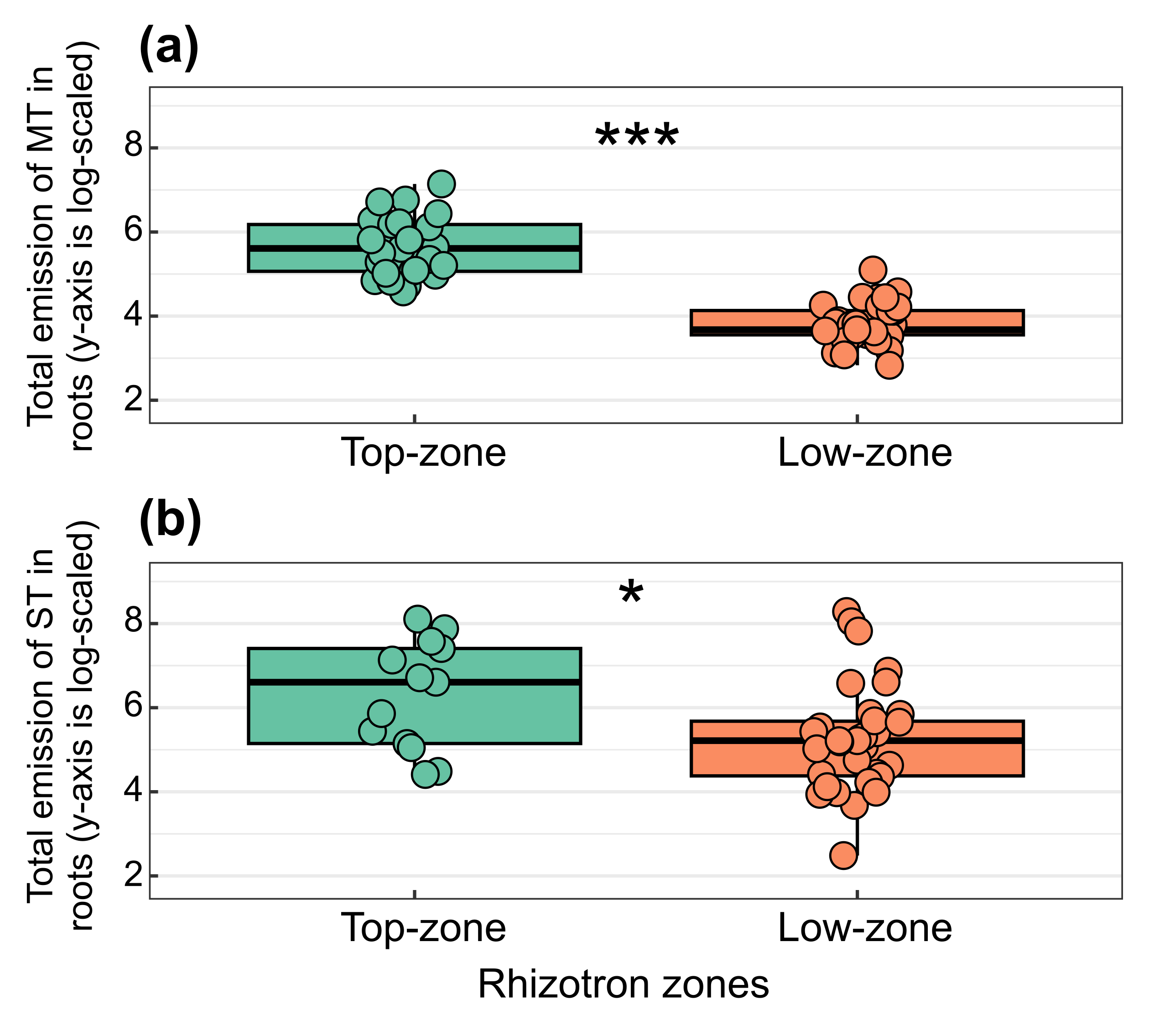

**Figure S5.** Root (a) monoterpenoid, and (b) sesquiterpenoid emissions across rhizotron zones: 1) top-zone – twister ~5 cm below the surface (near coarse roots); 2) low-zone – twisters on individual lower roots (30-40 cm from the top) enclosed with foil bags. The soil-background check showed no terpenoid peaks and was therefore omitted from the figure and analysis. Bonferroni-adjusted Dunn tests showed higher monoterpenoid and sesquiterpenoid emission in the top-zone relative to the low-zone (MT: ***; p < 0.001; SQT: *; p = 0.025). Positive sample sizes were: top-zone 25, low-zone 36 in panel a, and top-zone 13, low-zone 34 in panel b.


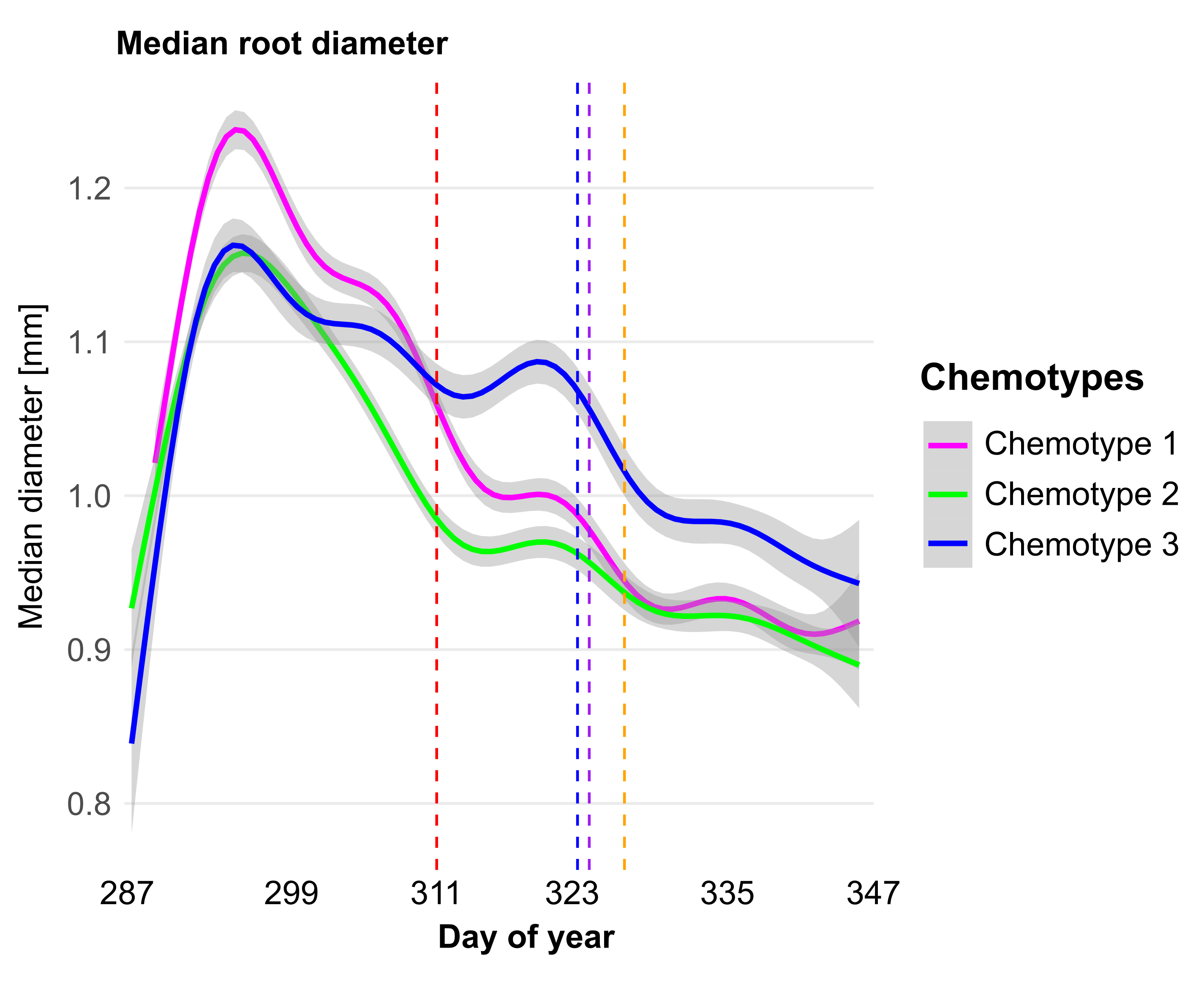


**Figure S6.** Median root diameter over the days of year (DOY 287–347) differed by chemotypes. Magenta, green, and blue curves show chemotype 1, chemotype 2, and chemotype 3. Vertical markers show, from left to right, the onset of wireworm treatment (day 313), aphid treatment (day 325), the rhizotron twister analysis (day 326), and the initial harvest (day 329).


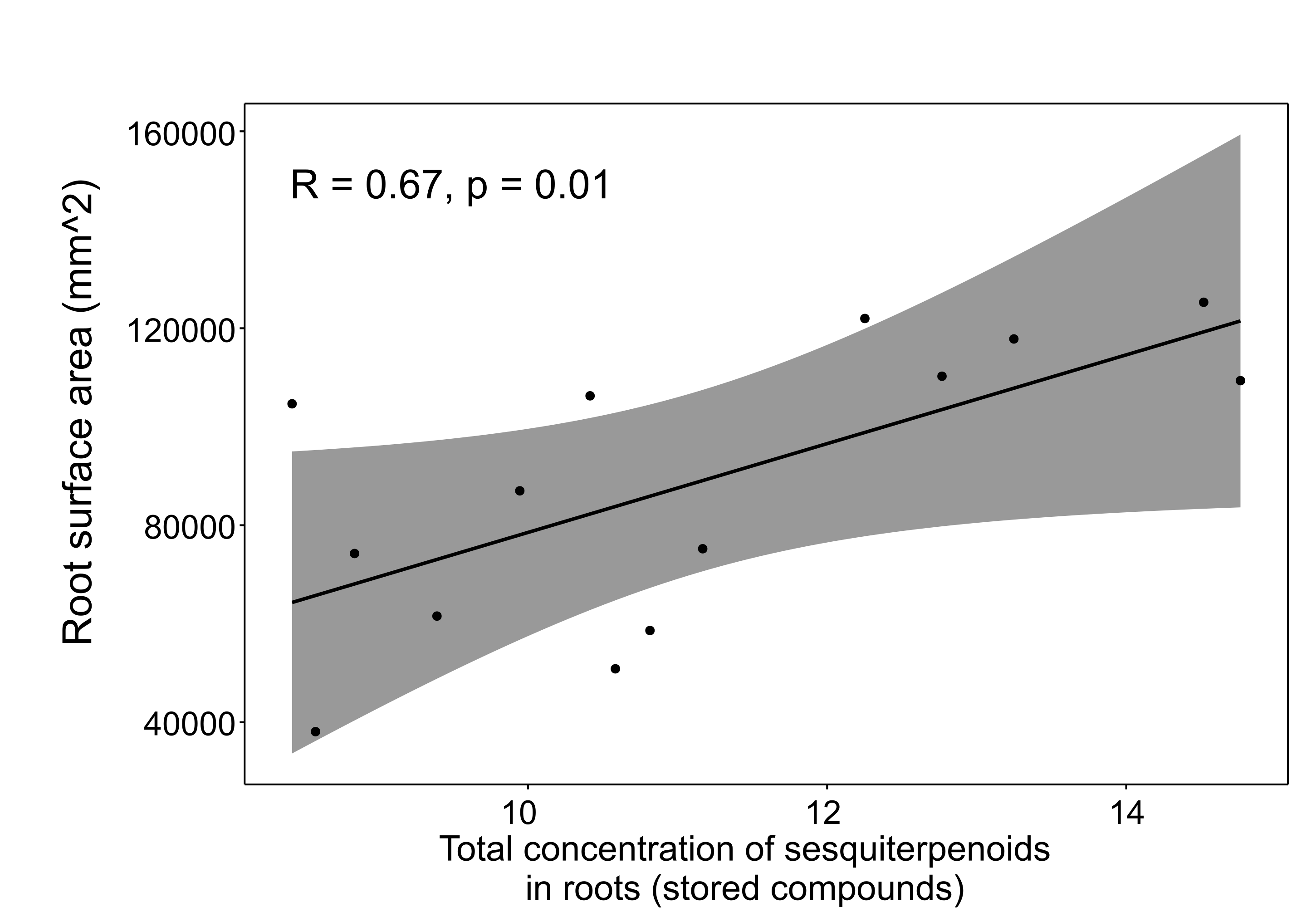


**Figure S7.** Root surface area (mm²) measured a week before the final harvest is plotted against the total concentration of stored sesquiterpenoids (Pearson’s R = 0.67, p = 0.01) in the roots of untreated plants (n = 14). The solid line shows the fitted linear regression with the 95% confidence interval (grey band).

| **Response** | **Comparison** | **Wald χ²** | **BH-adjusted p** |
| --- | --- | --- | --- |
| Surface area | Chemotype 1 − Chemotype 2 | **5.94** | **0.022*** |
|  | Chemotype 1 − Chemotype 3 | **9.40** | **0.007**** |
|  | Chemotype 2 − Chemotype 3 | 0.38 | 0.539 |
| Total root length | Chemotype 1 − Chemotype 2 | **4.94** | **0.039*** |
|  | Chemotype 1 − Chemotype 3 | **13.01** | **0.001**** |
|  | Chemotype 2 − Chemotype 3 | 1.83 | 0.176 |
| Volume | Chemotype 1 − Chemotype 2 | **6.80** | **0.027*** |
|  | Chemotype 1 − Chemotype 3 | *4.35* | *0.055.* |
|  | Chemotype 2 − Chemotype 3 | 0.26 | 0.611 |
| Root:shoot ratio | Chemotype 1 − Chemotype 2 | **11.49** | **0.001**** |
|  | Chemotype 1 − Chemotype 3 | **16.92** | **<0.001***** |
|  | Chemotype 2 − Chemotype 3 | 0.50 | 0.479 |

**Table S1.** Pairwise chemotype contrasts for root traits from generalized additive mixed models (GAMMs). Models were fit with mgcv using REML and included chemotype-specific smooths of day-of-year (factor-smooth, 𝑘 = 10) and a plant-level random intercept. Estimated marginal means were averaged uniformly over the observed time range. Pairwise differences were tested with **Wald** χ² (df = 1) from the EMM contrasts, and p-values were **Benjamini-Hochberg-adjusted within trait**. We limited statistical inference to data collected between day 287 and 329 (from the first measurement until the initial harvest) as this time window provided the most complete, uninterrupted observations.
